# A causal inference study exploring the impact of iron status on the risk of thyroid cancer based on two-sample mendelian randomization

**DOI:** 10.1007/s12672-025-02270-3

**Published:** 2025-04-07

**Authors:** Yihan Shi, Wenlian Zheng, Guanglun Yang, Hong Liu, Lei Xing

**Affiliations:** 1 Department of Breast and Thyroid Surgery, The First Affiliated Hospital of Chongqing Medical University, Chongqing, 400016 China; 2 Department of Burn and Wound Repair, Shaoguan First People’s Hospital, Shaoguan, 512023 Guangdong China

**Keywords:** Iron status, Thyroid cancer, Serum iron, Serum ferritin, Transferrin saturation, Mendelian randomization

## Abstract

**Background & aims:**

Thyroid cancer is prone to early lymph node metastasis.This study investigated the influence of iron status on thyroid cancer risk and its mediating role in the relationship between thyroid cancer incidence and thyroid cancer-related exposure factors.

**Method:**

Utilizing iron status-related Single Nucleotide Polymorphisms as instrumental variables, the research analyzed summary data on iron status and thyroid cancer from Genome-Wide Association Studies following the Two-sample Mendelian randomization guidelines, primarily using the Inverse-variance weighted method, with Mendelian randomization-Egger method, weighted median method, simple mode, and weighted mode as supplementary analyses. The reliability and robustness of the results were assessed using the Leave-one-out analysis and Cochran’s Q Test.

**Results:**

The findings indicate that the iron status has a vital causal relationship with the occurrence of thyroid cancer. The Inverse-variance weighted model results revealed Iron || id:ieu-a-1049: OR = 1.409, 95%CI = (1.043, 1.904), *P* < *0.05*; Ferritin || id:ieu-a-1050: OR = 2.029, 95% CI = (1.081, 3.808), *P* < *0.05*; Transferrin Saturation || id:ieu-a-1051: OR = 1.337, 95%CI = (1.058, 1.690), *P* < *0.05*. The reliability and robustness of these results were further supported by the Leave-one-out analysis and Cochran’s Q Test (*P* > *0.05*).

**Conclusion:**

The study establishes a certain causal link between iron status and thyroid cancer, indicating that transferrin saturation, serum ferritin and serum iron are associated with thyroid cancer incidence.

**Supplementary Information:**

The online version contains supplementary material available at 10.1007/s12672-025-02270-3.

## Introduction

Thyroid cancer (TC) is one of the most prevalent cancers, comprising four primary types: follicular cancer, papillary cancer, anaplastic cancer, and medullary cancer [1, 2]. Despite various treatment options and generally favorable postoperative outcomes, early-stage TC often results in cervical lymph node metastasis [1]. Studies indicate that 30% to 80% of patients with papillary thyroid cancer (PTC) experience cervical lymph node metastasis, particularly affecting the central compartment (Zone VI) lymph nodes in about 33% of cases [1]. Recurrence and metastasis notably decrease the survival rate of patients with TC [1, 3, 4]. Currently, effective early diagnostic markers and treatment methods for recurrent and extensively metastatic TC are lacking.

Iron is vital for cell growth and division. Recent studies have connected iron metabolism disorders to breast cancer progression, invasiveness, and recurrence [5]. Elevated iron levels correlate with cancer incidence and mortality rates [6]. Clinically, iron status is assessed via serum iron, ferritin, transferrin, and transferrin saturation [7]. Iron, as an important component of human enzymes and proteins, plays a crucial role in transporting oxygen, DNA synthesis and repair, energy metabolism, cellular respiration, immune response and signal transduction. However, iron is a lively element, and excessive iron could induce oxidative stress, forming reactive oxygen species and lipid peroxidation products [8]. Ferritin, a key iron storage protein, is vital for iron homeostasis and plays roles in cell proliferation, iron transport, immune suppression, and angiogenesis. Ferritin is overexpressed in many cancer cells, and the survival and drug resistance of cancer cells are associated with its elevated levels [9]. Higher serum ferritin levels correlate with more invasive disease and worse clinical outcomes [10]. Transferrin, as the main plasma protein for iron transport and an acute phase reactant, is a key marker of iron homeostasis in the blood, similar to ferritin. Transferrin levels decrease during in iron overload, malignancy, and inflammation, while they increase in cases of iron deficiency [8]. Transferrin saturation, indicating circulating iron availability [11], has been associated with higher cancer risk and mortality when levels exceed 40% above normal reserves [12]. Iron status is linked to various malignancies, including prostate [13], gastric [14], ovarian [8], and breast cancers [5].

Current study on the correlation between iron status and TC is restricted and inconclusive regarding iron’s role in the germination and development of TC. In order to bridge this gap, a Mendelian randomization (MR) analysis was performed to ascertain the causal relationship between biomarkers of iron status and TC. The objective of this analysis was to uncover novel strategies for prevention and treatment. MR, as a tool variable that utilizes genetic variation, is commonly used to assess causal relationships in observational data and to infer the correlation between outcome and exposure.

## Materials and methods

### Research report guidelines and research design

**Fig. 1:**
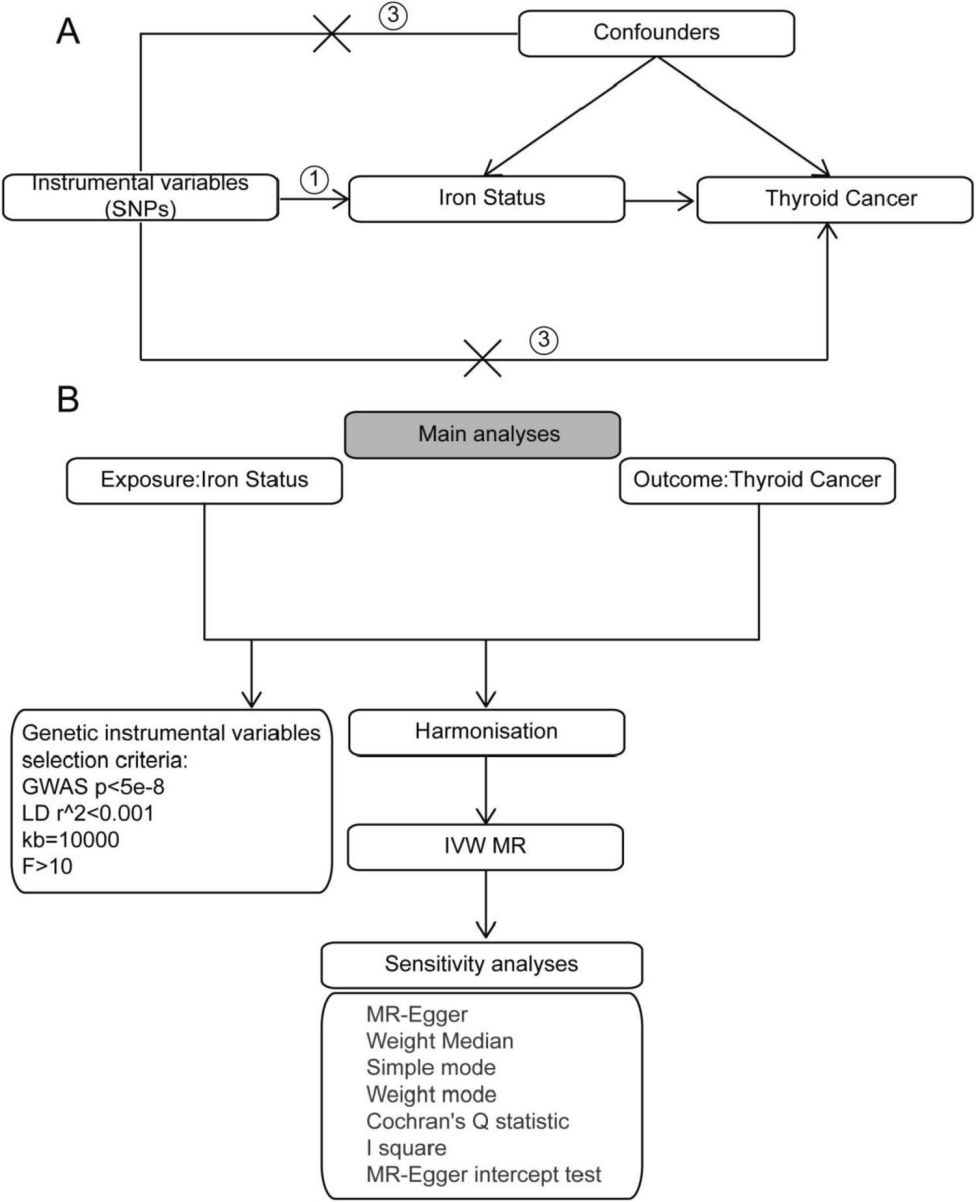
Technical roadmap

This study employs two-sample Mendelian randomization (TSMR) alongside publicly accessible datasets to investigate the influence of iron status on the prevalence of TC. Additionally, it examines the mediating effect of iron status in the association between exposure factors related to TC and its incidence. The research report follows the MR reporting guidelines, specifically the Strengthening the Reporting of Observational Studies in Epidemiology Using Mendelian Randomization (STROBE-MR Statement) [15], with the study design schematic depicted in Fig. 1.

### Data source

The iron status GWAS (Genome-Wide Association Study) data is sourced from a study by Beben Benyamin et al. [16], based on samples from the European population. Summary statistics regarding the association of iron status were collected and subsequently standardized utilizing the R package TSMR. Similarly, the TC GWAS data comes from a study by Sakaue S et al. [17], also utilizing European population samples. The summary statistics for the association of TC were standardized utilizing the identical R package.

### Selection of instrumental variables

Effective genetic instrumental variables must meet three core assumptions: firstly, relevance, indicating that there must be a significant correlation between the exposure factors and the instrumental variables chosen for the study; secondly, independence, meaning potential confounding factors that may affect exposure or outcome must not have a significant relationship with the instrumental variable; and thirdly, exclusion restriction, stipulating that the result can only be influenced by the instrumental variable through the path "instrumental variable → exposure → result."

In the assessment of univariate MR concerning the influence of iron status on TC, as well as the reverse-causal MR analysis regarding the effect of TC on iron status, and the multivariate MR analysis that incorporates both iron status and thyroid cancer-related exposure factors, specific criteria were established for the selection of instrumental variables. The screening criteria mandated that Single Nucleotide Polymorphisms (SNPs) from GWAS exhibit a significance level of P < 5 × 10^^−8^. Additionally, SNPs that were in linkage disequilibrium—defined as those with an *r* value less than 0.001 and a physical distance exceeding 10,000 kilobases between any two genes—were systematically excluded from consideration. Instrumental variables derived from GWAS pertaining to the outcome data were obtained based on the chosen SNPs. The *F*-statistics were computed to evaluate the potential bias introduced by weak instrumental variables. An *F*-statistic less than 10 suggests that the genetic variation is a weak instrumental variable, potentially introducing bias into the results [18]. To mitigate this, weak instruments were excluded. The formula for calculating the *F*-statistic is as follows:

$$\text{F}=\frac{\text{N}-\text{k}-1}{\text{k}}\times \frac{{\text{R}}^{2}}{1-{\text{R}}^{2}}$$

In this context, *n* denotes the sample size, *k* represents the count of instrumental variables employed, and *R*^*2*^ indicates the degree to which these instrumental variables account for the exposure. The expression for *R*^*2*^ is articulated as follows: $R^{2} = 2 \times \left( {1 - MAF} \right) \times MAF \times 2\beta$, where MAF signifies the minimum allele frequency and *β* denotes the effect size of the allele.

### Estimates of causal effects derived from MR analyses

Multiple TSMR methods were utilized to assess the causal effect of iron status on TC, including Weighted Mode methods, Simple Mode, Weighted Median, MR-Egger and Inverse-Variance Weighted (IVW). Research indicates that the IVW method often outperforms others under certain conditions [19]; This method’s framework omits the intercept term in regression analysis and employs the inverse of the outcome variance as a weighting factor during the fitting process. As a result, in the absence of pleiotropic influences and regardless of any variations, the IVW approach was utilized as the principal method for MR analysis, supported by four supplementary techniques. In instances where heterogeneity was observed, the IVW random effects model was utilized, while the MR-Egger method was employed to derive results in situations where pleiotropy was present.

### Sensitivity analysis

The analysis involved heterogeneity testing, pleiotropy inspection, and sensitivity analysis, detailed as follows: firstly, Heterogeneity Assessment: The Cochran’s Q test was utilized to evaluate the heterogeneity present among SNP estimates. A statistically significant result from the Cochran’s Q test suggests considerable heterogeneity, thereby necessitating the application of the IVW random effects model to accurately estimate the causal effect size in instances of pronounced heterogeneity. Given that Cochran’s Q test solely detects the existence of heterogeneity without assessing its distribution, the *I*^*2*^ statistic was utilized to represent the fraction of heterogeneity within the overall variation of instrumental variables. The classification of *I*^*2*^ is delineated as follows:* I*^*2*^ ≤ *0* (assigned a value of 0, signifying the absence of observed heterogeneity), *I*^*2*^ ranging from 0 to 25% (indicative of mild heterogeneity), *I*^*2*^ between 25 and 50% (representing moderate heterogeneity), and *I*^*2*^ exceeding 50% (denoting high heterogeneity). The precise formula for calculation is presented below:

$${\text{I}}^{{2}} = \frac{Q - df}{Q} \times {1}00\%$$

Secondly, Pleiotropy Assessment: The MR-Egger approach was utilized to evaluate pleiotropy within the instrumental variables. A *P* value below 0.05 for the MR-Egger intercept suggests significant horizontal pleiotropy associated with genetic variation.

Thirdly, Leave-One-Out Test: This assessment computed the MR outcomes of the residual instrumental variables by systematically omitting each SNP to ascertain whether any particular SNP had an effect on the relationship between iron status and TC. A substantial difference between the MR effect estimates and the total effect estimates after excluding an instrumental variable suggests that the MR effect estimates are sensitive to that specific SNP.

### Statistic analysis

All statistical analyses and data calculations were performed using R programming (https://www.r-projec t.org/, version 4.2.2). The TSMR package facilitated the MR analysis [20]. The reliability and robustness of the results were evaluated using leave-one-out analysis and the Cochran’s Q test, while the MR-Egger intercept method tested for horizontal pleiotropy. For TC MR analysis, the evaluation indices included the 95% confidence interval (95% CI) and the odds ratio (OR). All statistical *P* values were two-sided, with a *P* value of less than 0.05 considered statistically significant.

## Results

### Technical roadmap

A. The schematic design of MR analysis illustrates its underlying assumptions: firstly, the correlation assumption posits that the chosen instrumental variables must be vitally related to the exposure factors; secondly, the principle of independence asserts that the instrumental variable must not exhibit a vital correlation with any possible confounding factors that could affect either the exposure or the outcome; thirdly, the exclusivity assumption posits that the instrumental variable affects the outcome solely via the route "instrumental variable → exposure."

B. The diagram illustrating the analytical methodologies employed in this study. LD, linkage disequilibrium; GWAS, genome-wide association study; MR, Mendelian randomization; IVW, inverse variance weighted; SNP, single nucleotide polymorphism.

### An examination of the causal link between TC and iron status

Data from the Opengwas database (https://gwas.mrcieu.ac.uk/) was exploited to select relevant SNP loci for iron status, providing the preliminary causal analysis results. Subsequent to the alignment of this dataset with the GWAS data pertaining to TC (ebi-a-GCST90018929), the effect sizes corresponding to all instrumental variables were extracted. Following the harmonization process, only the instrumental variables that demonstrated a correlation with both TC and iron status were incorporated into the MR analysis. Table 1 illustrates the distinct instrumental variables associated with factors influencing iron status exposure. Instrumental variables with an *F*-test statistic greater than 10 were retained, indicating that most SNPs screened in this study were strong-effect instrumental variables, thereby limiting the potential bias from weak instrumental variables.

**Table 1:** Screening of instrumental variable and *F* test for instrumental variable strength in iron status and TC

Exposure	Outcome	Number of SNPs	Median of *F*	Minimum of *F*	Maximum of *F*
Ferritin || id:ieu-a-1050	Thyroid cancer || id:ebi-a-GCST90018929	4	45.971	30.307	127.305
Iron || id:ieu-a-1049	Thyroid cancer || id:ebi-a-GCST90018929	3	342.038	50.079	346.675
Transferrin Saturation || id:ieu-a-1051	Thyroid cancer || id:ebi-a-GCST90018929	4	222.728	35.763	808.396

*SNP* single nucleotide polymorphism, *TC* thyroid cancer

### MR causal effect estimation

**Fig. 2:**
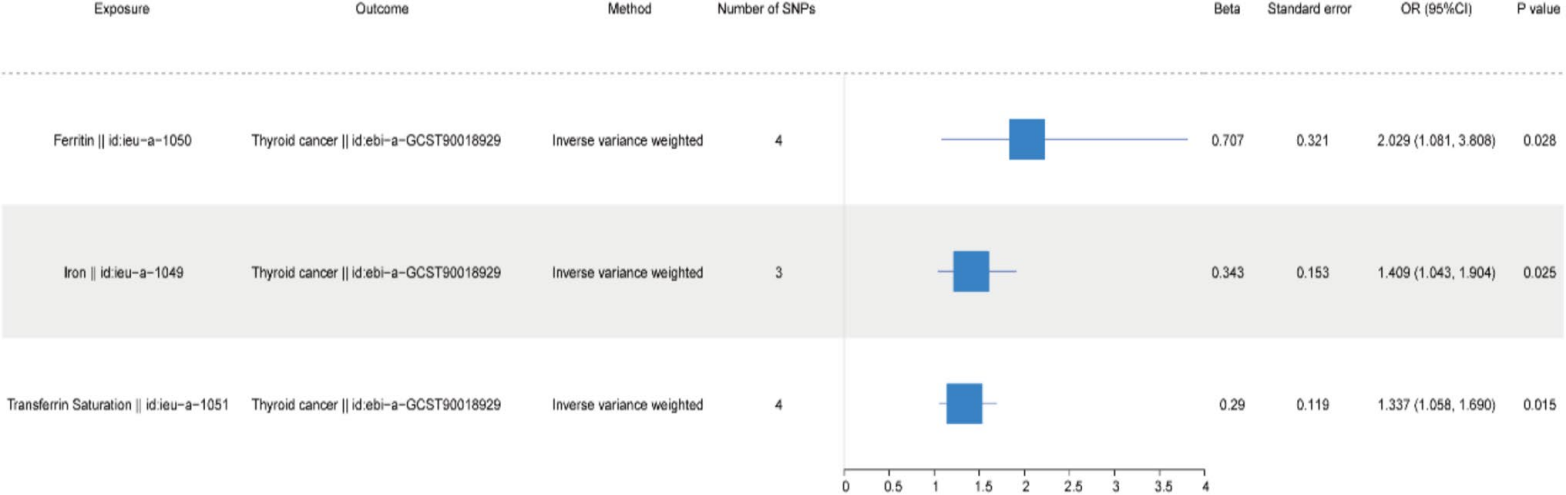
Forest plot of MR results for iron status and TC. *CI* confidence interval, *OR* odds ratio, *SNPs* single nucleotide polymorphism, *TC* thyroid cancer

The analysis employed MR Egger, Weighted Mode methods, Simple Mode, IVW and Weighted Median. The IVW model outcomes indicated a vital causal relationship between iron status (ieu-a-1049 for iron, ieu-a-1050 for ferritin, and ieu-a-1051 for transferrin saturation) and TC (Thyroid Cancer: ebi-a-GCST90018929), with *p* values less than 0.05 (see Table 2). Detailed information about these three iron status markers is provided in Table S1. The IVW model results demonstrate that increases in ieu-a-1050 (Ferritin), ieu-a-1049 (Iron), and ieu-a-1051 (Transferrin Saturation) could cause TC (Thyroid Cancer: ebi-a-GCST90018929) increased the risk of TC. The results of the MR analysis of Iron Status and TC (ebi-a-GCST90018929) are presented in a forest plot (Fig. 2).

**Table 2:** Results of MR analysis of iron status on the incidence of TC

Exposure	Outcome	Method	Number of SNPs	*β*	Standard error	OR(95%CI)	*P* value
Ferritin || id:ieu-a-1050	Thyroid cancer || id:ebi-a-GCST90018929	Inverse variance weighted	4	0.707	0.321	2.029 (1.081, 3.808)	0.028
Ferritin || id:ieu-a-1050	Thyroid cancer || id:ebi-a-GCST90018929	MR Egger	4	0.086	0.605	1.090 (0.333, 3.567)	0.9
Ferritin || id:ieu-a-1050	Thyroid cancer || id:ebi-a-GCST90018929	Simple mode	4	1.394	0.589	4.031 (1.271, 12.786)	0.099
Ferritin || id:ieu-a-1050	Thyroid cancer || id:ebi-a-GCST90018929	Weighted median	4	0.641	0.387	1.899 (0.890, 4.052)	0.097
Ferritin || id:ieu-a-1050	Thyroid cancer || id:ebi-a-GCST90018929	Weighted mode	4	0.568	0.441	1.765 (0.743, 4.189)	0.288
Iron || id:ieu-a-1049	Thyroid cancer || id:ebi-a-GCST90018929	Inverse variance weighted	3	0.343	0.153	1.409 (1.043, 1.904)	0.025
Iron || id:ieu-a-1049	Thyroid cancer || id:ebi-a-GCST90018929	MR Egger	3	0.566	0.313	1.761 (0.954, 3.251)	0.321
Iron || id:ieu-a-1049	Thyroid cancer || id:ebi-a-GCST90018929	Simple mode	3	0.384	0.199	1.469 (0.995, 2.169)	0.193
Iron || id:ieu-a-1049	Thyroid cancer || id:ebi-a-GCST90018929	Weighted median	3	0.384	0.156	1.469 (1.082, 1.994)	0.014
Iron || id:ieu-a-1049	Thyroid cancer || id:ebi-a-GCST90018929	Weighted mode	3	0.385	0.184	1.470 (1.025, 2.110)	0.172
Transferrin Saturation || id:ieu-a-1051	Thyroid cancer || id:ebi-a-GCST90018929	Inverse variance weighted	4	0.29	0.119	1.337 (1.058, 1.690)	0.015
Transferrin Saturation || id:ieu-a-1051	Thyroid cancer || id:ebi-a-GCST90018929	MR Egger	4	0.272	0.21	1.312 (0.870, 1.979)	0.325
Transferrin Saturation || id:ieu-a-1051	Thyroid cancer || id:ebi-a-GCST90018929	Simple mode	4	0.246	0.165	1.279 (0.927, 1.766)	0.231
Transferrin Saturation || id:ieu-a-1051	Thyroid cancer || id:ebi-a-GCST90018929	Weighted median	4	0.288	0.126	1.333 (1.041, 1.708)	0.023
Transferrin Saturation || id:ieu-a-1051	Thyroid cancer || id:ebi-a-GCST90018929	Weighted mode	4	0.283	0.144	1.327 (1.001, 1.759)	0.144

*β* Mendelian randomization analysis effect coefficient, *OR* odds ratio, *CI* confidence interval, *SNP* single nucleotide polymorphism, *MR* mendelian randomization, *TC* thyroid cancer

**Fig. 3:**
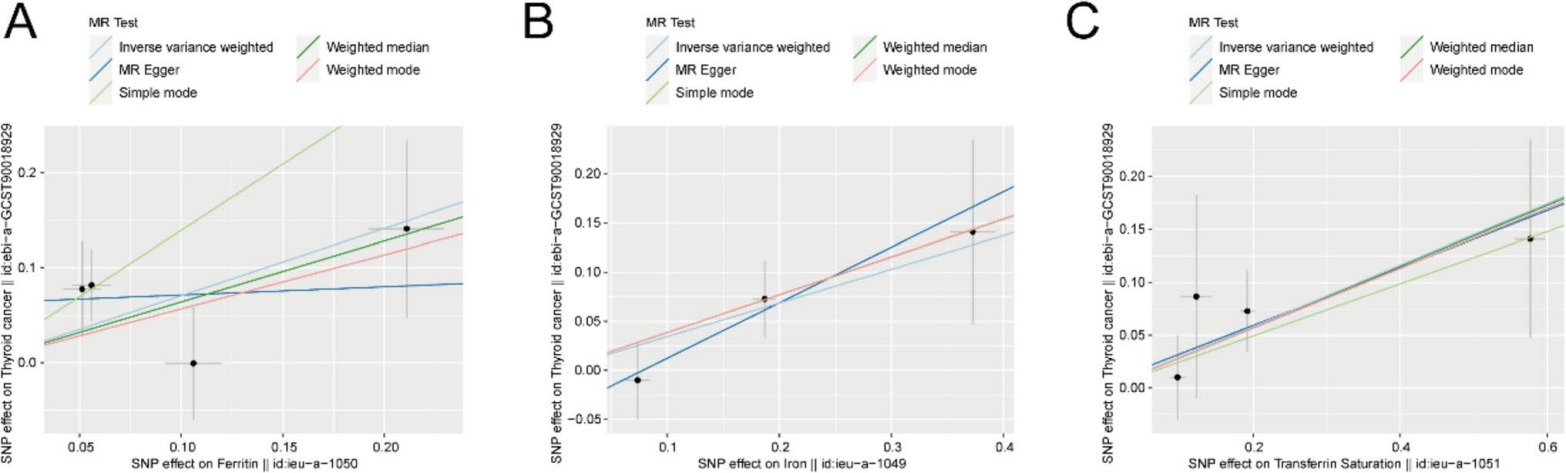
Estimates of effects derived from various models for MR analysis concerning total TC and iron status. **A**–**C** Scatter plots illustrating the causal relationship between iron status (ieu-a-1050 Ferritin, ieu-a-1049 Iron, ieu-a-1051 Transferrin Saturation) and TC (ebi-a-GCST90018929). The gradient of the line indicates the strength of the causal association anticipated by the various models. *MR* Mendelian randomization, *SNPs* single nucleotide polymorphism, *IVW* Inverse-Variance Weighted

Figure 3 illustrates the analysis of three iron status variables using five models (MR Egger, Weighted Mode, Simple Mode, IVW and Weighted Median). The outcomes indicate that the effect estimates from the Simple Mode, Weighted Mode and Weighted Median align closely with the direction and orientation of the IVW model.

### Sensitivity analysis

The Cochran’s Q test was employed to assess heterogeneity in the relationship between three iron status indicators and TC (ebi-a-GCST90018929) (Table 3). The results indicated no significant heterogeneity (Cochran’s Q *p*-value > 0.05).

**Table 3:** Heterogeneity results of the Cochran’s Q test for MR analysis of iron status on the incidence of TC

Exposure	Outcome	Method	Cochran Q	Cochran Q df	Cochran Q p-value	*I*^*2*^ (%)
Iron || id:ieu-a-1049	Thyroid cancer || id:ebi-a-GCST90018929	Inverse variance weighted	0.864026	2	0.649201	0
Ferritin || id:ieu-a-1050	Thyroid cancer || id:ebi-a-GCST90018929	Inverse variance weighted	3.563626	3	0.312603	15.81607
Transferrin Saturation || id:ieu-a-1051	Thyroid cancer || id:ebi-a-GCST90018929	Inverse variance weighted	0.761118	3	0.858743	0

*Q* Cochran Q test statistic, Q df, Q tests the degrees of freedom, *MR* mendelian randomization, *TC* thyroid cancer

Additionally, the MR Egger regression was operated to inspect pleiotropy at the instrumental variable level for the causal effect of three iron status indicators on TC (ebi-a-GCST90018929). The results indicated that the intercept’s statistical hypothesis test p-values were greater than 0.05, and the intercept values were close to zero. This suggests that the causal inference between TC occurrence and iron status in this study was not influenced by pleiotropy (Table 4).

**Table 4:** Horizontal pleiotropy test results for MR analysis of iron status on the incidence of TC

Exposure	Outcome	MR-Egger intercept	Standard error	*P* value
Iron || id:ieu-a-1049	Thyroid cancer || id:ebi-a-GCST90018929	− 0.04434	0.054259	0.563853
Ferritin || id:ieu-a-1050	Thyroid cancer || id:ebi-a-GCST90018929	0.062858	0.05306	0.357846
Transferrin Saturation || id:ieu-a-1051	Thyroid cancer || id:ebi-a-GCST90018929	0.004919	0.045022	0.922977

*MR-Egger* mendelian randomization-Egger, *TC* thyroid cancer

**Fig. 4:**
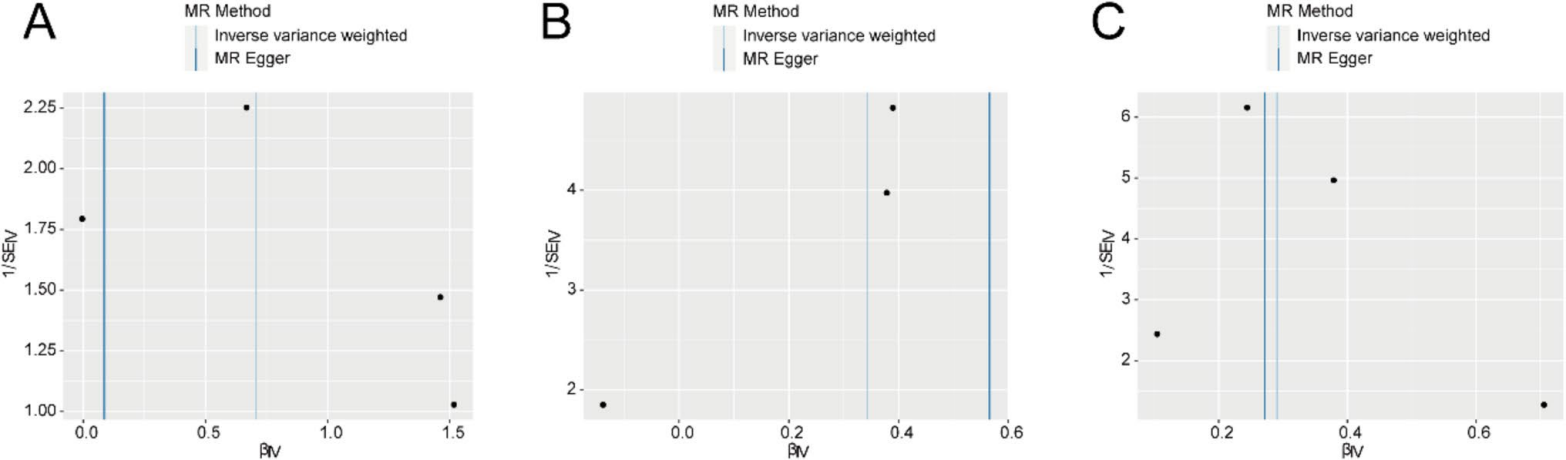
Funnel plot of heterogeneity test for MR analysis of iron status and TC. **A**–**C** Funnel plots for heterogeneity tests of iron status (ieu-a-1050 Ferritin, ieu-a-1049 Iron, ieu-a-1051 Transferrin Saturation) and TC (ebi-a-GCST90018929). The causal effect estimates derived from the IVW method and the MR Egger model are represented by lines

The instrumental variable funnel plots (Fig. 4A–C) illustrate that the scatter basic distribution of the causal effect of iron status on TC (ebi-a-GCST90018929) is symmetric, indicating no potential bias in the results.

**Fig. 5:**
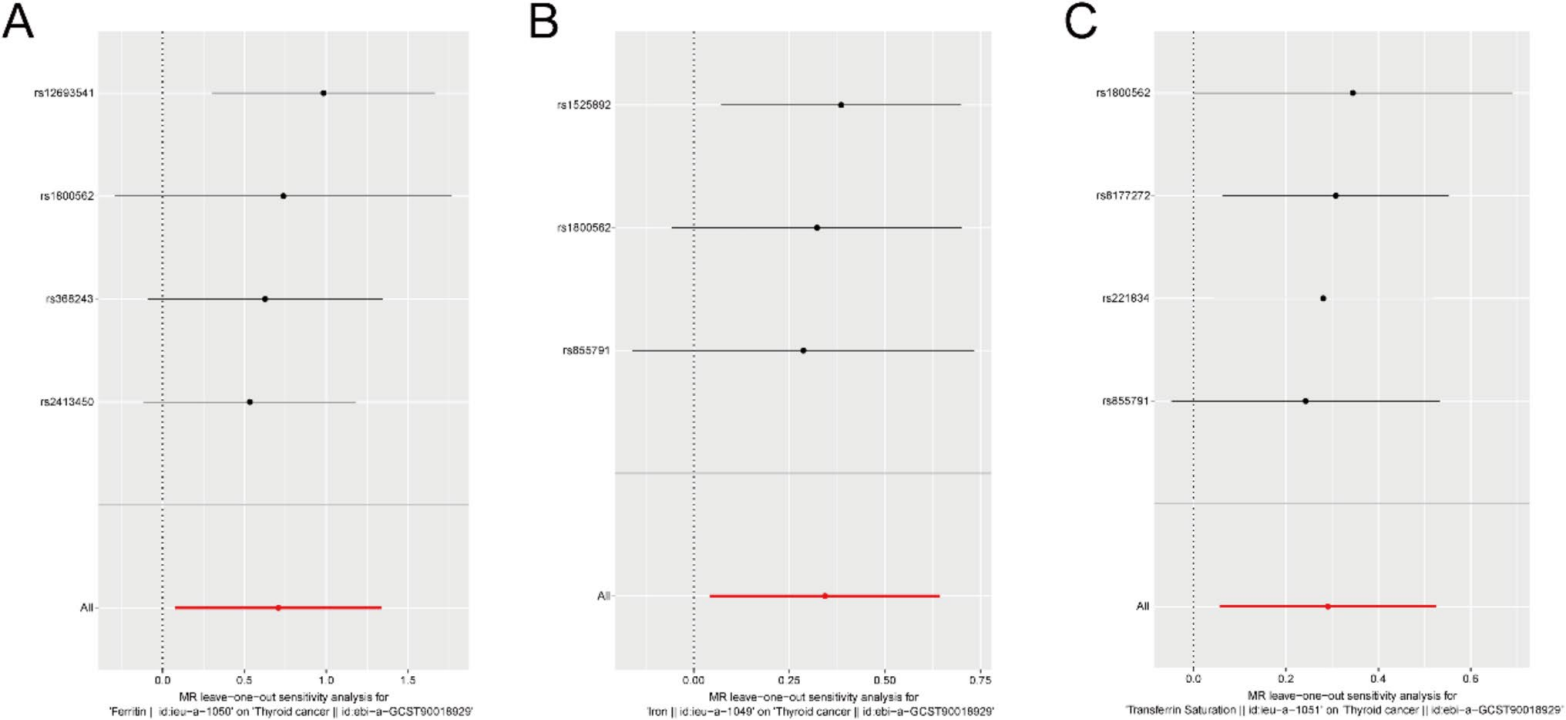
MR leave-one-out analysis of iron status and TC. **A**–**C** Iron status ieu-a-1050 (Ferritin), ieu-a-1049 (Iron), MR leave-one-out analysis of ieu-a-1051 (Transferrin Saturation) and TC ebi-a-GCST90018929. *MR* Mendelian randomization, *IVW* Inverse-Variance Weighted, *TC* thyroid cancer

The leave-one-out analysis examined the causal effect of iron status on TC (ebi-a-GCST90018929) by sequentially removing each instrumental variable locus. The results demonstrated that the overall effect of the instrumental variable set was not significantly biased (Fig. 5).

### Assessment of the impact of iron status on the risk of TC

**Fig. 6:**
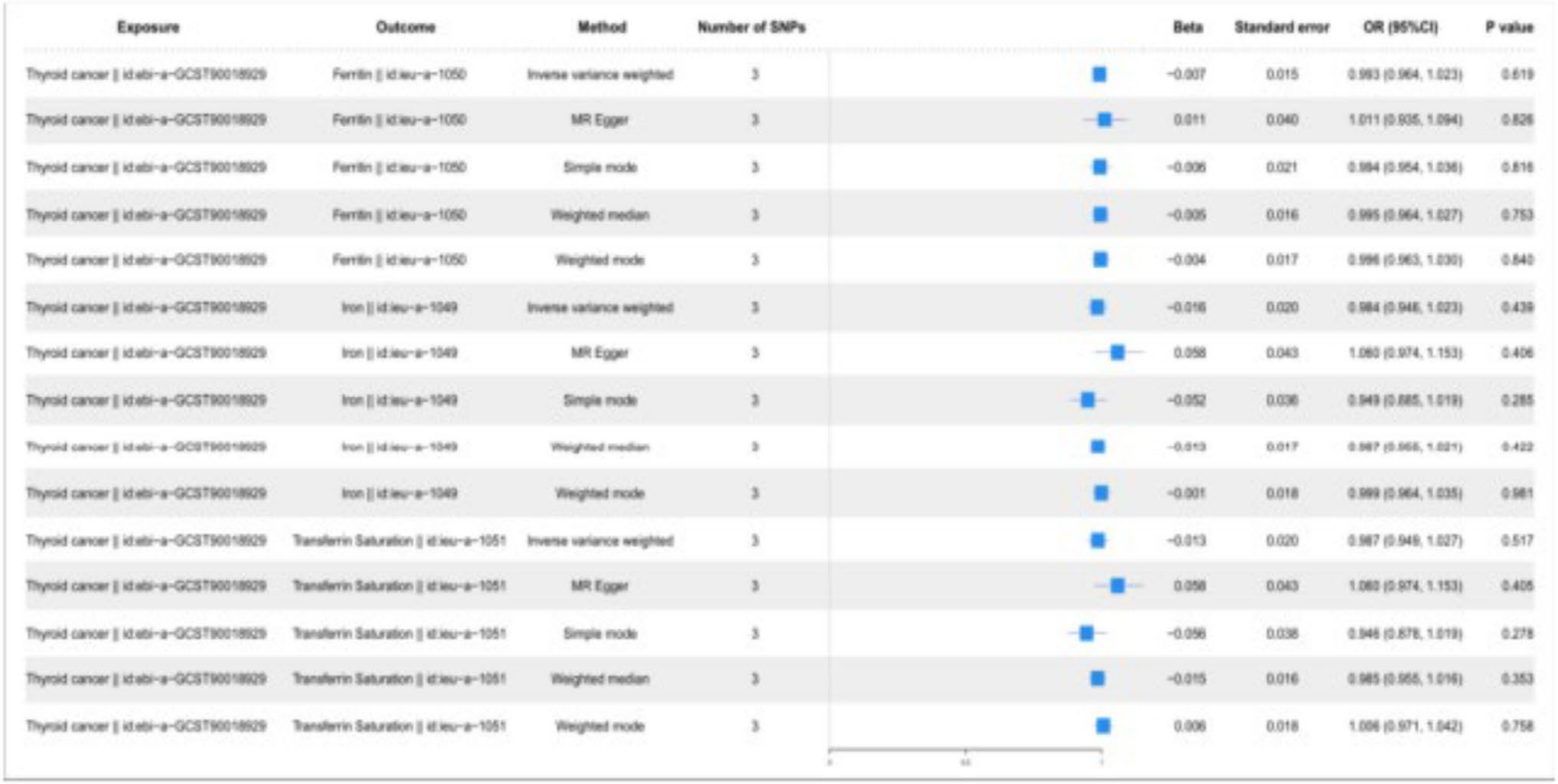
Forest plot of MR results for TC and iron status. *SNPs* single nucleotide polymorphisms, *OR* odds ratio, *CI* confidence interval, *MR* Mendelian randomization, *TC* thyroid cancer

In our GWAS for TC (ebi-a-GCST90018929), SNPs with P < 5e-8 were initially screened, and those in linkage disequilibrium (LD-r^2^ < 0.001 and physical distance between each pair of genes > 10,000 kb) were excluded. GWAS data for the three iron status categories were matched to obtain effect sizes for all instrumental variables, which were then processed through harmonization. Subsequently, analyses were conducted using Weighted Mode models, Simple Mode, IVW, Weighted Median and MR Egger (Fig. 6). The outcomes indicated that TC (ebi-a-GCST90018929) had no significant effect on iron status, including ieu-a-1049 (iron), ieu-a-1050 (ferritin), and ieu-a-1051 (transferrin saturation) (*P* > *0.05*). This advised that there is no significant causal relationship between TC (ebi-a-GCST90018929) and iron status.

## Discussion

Most patients with TC have a good prognosis post-surgery but are prone to cervical lymph node metastasis [1, 2]. It is essential to find effective early diagnostic markers and treatment methods to address lymph node metastasis in TC. Our research is making such efforts. MR methods have been employed in this research to examine the relationship between iron metabolism and TC by assessing biomarkers related to iron metabolism, aiming to provide new therapeutic strategies. The MR analysis method effectively controls for confounding factors, enhancing the reliability of the results.

This will further support the rationality of the instrumental variables and the robustness of the study. The specific calculation formulas are as follows:

$${\text{Ferritin }}\left( {{\text{ieu}} - {\text{a}} - {1}0{5}0} \right):{\text{ R}}^{{2}} = {\text{XX}},{\text{ Overall F}} - {\text{statistic}} = {\text{XX}}$$

$${\text{Iron }}\left( {{\text{ieu}} - {\text{a}} - {1}0{49}} \right):{\text{ R}}^{{2}} = {\text{XX}},{\text{ Overall F}} - {\text{statistic}} = {\text{XX}}$$

$${\text{Transferrin Saturation }}\left( {{\text{ieu}} - {\text{a}} - {1}0{51}} \right):{\text{ R}}^{{2}} = {\text{XX}},{\text{ Overall F}} - {\text{statistic}} = {\text{XX}}$$

The overall F-statistics are far above the standard threshold for weak instrumental variables (F > 10), indicating that the instrumental variables included in this study have sufficient explanatory power and statistical efficiency. This ensures the robustness of the results obtained through Mendelian randomization analysis.

The study yielded the following key findings: The IVW model outcomes demonstrated a vital causal relationship between iron status (ieu-a-1049: Iron), (ieu-a-1050: Ferritin), (ieu-a-1051: Transferrin Saturation) and the occurrence of TC (Thyroid Cancer: ebi-a-GCST90018929) (*p* < *0.05*). Specifically: Iron || id: ieu-a-1049: OR = 1.409, 95% CI = (1.043, 1.904), *P* = *0.025*; Ferritin || id: ieu-a-1050: OR = 2.029, 95% CI = (1.081, 3.808), *P* = *0.028*; Transferrin Saturation || id: ieu-a-1051: OR = 1.337, 95% CI = (1.058, 1.690), *P* = *0.015*. These outcomes indicate that increases in iron, serum ferritin, and transferrin saturation are relevant with an elevated risk of developing TC (ebi-a-GCST90018929). Sensitivity analyses confirmed the stability of these results, showing no significant heterogeneity or pleiotropic effects. These outcomes advise that iron status perhaps a critical venture factor for TC. Therefore, further exploration of the mechanisms underlying the development of TC from the perspective of iron status is warranted, alongside the development of new tactics for disease treatment and prevention.

In this study, stringent criteria were applied for the selection of instrumental variables (IVs), including an F-statistic greater than 10, a GWAS P-value less than 5e-8, and linkage disequilibrium (LD) thresholds (r^2^ < 0.001, kb < 10,000), to ensure the strength and reliability of the instrumental variables. As mentioned in reference [47], the inverse-variance weighted Mendelian randomization (IVW-MR) is the most widely used method, leveraging summary statistics from genome-wide association studies (GWAS) to infer the presence and strength of causal effects between exposures and outcomes. The use of weak instrumental variables may lead to inaccurate estimates. An F-statistic greater than 10 helps mitigate the weak instrument bias, as it indicates a sufficiently strong association between the instrumental variables and the exposure. Therefore, this study did not individually assess the strength of association between each SNP and iron status (exposure), which might, to some extent, affect the representativeness of the instrumental variables.From our analysis, increased iron status (iron, ferritin, and transferrin saturation) may elevate the risk of thyroid cancer, while reverse MR analysis did not reveal a significant effect of thyroid cancer on iron status, further strengthening the directionality of the causal inference. Additionally, we employed multiple MR analysis methods, including inverse-variance weighted (IVW), MR-Egger, weighted median, and others, to ensure the robustness of the estimates. Sensitivity analyses, such as heterogeneity tests, pleiotropy tests, and leave-one-out analyses, were conducted to evaluate the reliability of the results. Although the current study has limitations in single-SNP-level analysis, the consistency across multiple methods and the results of sensitivity analyses support the main conclusions of this study.

Recurrence and metastasis significantly decrease survival rates [3]. Iron metabolism, a current research hotspot, has been shown in experiments that chronic imbalance of redox balance caused by high intracellular iron concentrations might regulate specific signaling networks associated with malignant tumors [5].

Iron, an essential component of proteins and enzymes, plays pivotal roles in transporting oxygen, DNA synthesis and repair, energy metabolism, cellular respiration, immune response and signal transduction [8]. Iron metabolism occurs in three stages: absorption, storage, and excretion [21]. Proper intracellular iron balance is usually maintained by circulating glycoprotein transport proteins, which transport extracellular iron, and ferritin complexes, which store and transport imported iron. In mammals, iron export proteins regulate the efflux of iron and may export intracellular iron [22, 23].

With high intracellular iron concentration, most carcinoma cells represent anomalous iron metabolism [21]. Excessive iron, a highly reactive element, can induce oxidative stress, leading to the shape of reactive oxygen species and lipid peroxidation outcomes, ultimately resulting in cell death [8]. There is a correlation between iron levels and cancer incidence and mortality rates [7].

J. Alonso García and colleagues conducted a comparative assessment of Fe metal groups in two different malignant tumor breast cancer cell lines, MDA-MB-231 and MCF-7, and found that total iron concentration is significantly higher in the lower malignancy phenotype. Significant differences in ferritin molecule iron content between the cell lines suggest that in the most malignant phenotype, ferritin functions beyond iron storage, highlighting its potential as a cancer biomarker [5]. Our research also found a correlation between iron and ferritin and TC.

Transferrin and ferritin complexes are involved in the transport and deposition of iron. Epidemiological studies have established an association between changes in carcinoma and iron metabolism [8]. Ferritin, composed of two subunits: heavy chain and light chain, binds and stores iron [24–26]. The subunits play different roles: ferritin heavy chain (FHC) has ferrous oxidase activity, facilitating rapid iron absorption and release, while ferritin light chain (FTL) aids in long-term iron storage [27].

Recent studies have shown that FHC is also involved in many cellular regulatory pathways, for instance cell proliferation [28], chemokine signaling [29], and angiogenesis [30]. FHC also regulates cellular functions by physically interacting with other molecules. Under oxidative stress, FHC could bind to and transcriptionally activate the p53 protein [31]. Similarly, FHC interacts with the chemokine receptor CXCR4, downregulating signaling pathways triggered by its ligand CXCL12. Given that CXCR4 is highly expressed in various human tumors, FHC’s ability to regulate the CXCR4 pathway might partially elucidate its complex role in tumor transformation [29].

FHC influences the expression of various cancer-related genes, including p53 [32], E1A [33],cMyc [34], and c-Jun [35]. Research by Di Sanzo et al. demonstrated that shRNA interference to downregulate FHC significantly reduces melanoma cell proliferation both in vivo and in vitro [36]. In the K562 leukemia cell line, FHC could regulate the expression of specific oncogenic miRNAs [37, 38].

The source of serum ferritin remains uncertain. Previous studies have shown that tumor-associated macrophages (TAM) produce and secrete ferritin [39, 40]. High ferritin levels in TAM might guard them from iron-induced harm, thereby promoting survival, angiogenesis, and proliferation [41]. Additionally, TAM-secreted ferritin might directly support and maintain tumor proliferation. Buranrat et al. found that MCF-7 breast cancer cells have been activated by ferritin in a concentration- and time-dependent manner. Elevated serum and tissue ferritin levels are associated with cancer [10], with higher ferritin levels associating with more invasive disease and inferior clinical results [10]. However, a study by Nadia Lobello et al. found that low FHC expression levels correlate with shorter survival times. In this study, FHC gene silencing in SKOV3 cells significantly enhanced cell viability and induced more aggressive behavior [38]. The mechanisms by which ferritin inhibits or promotes tumors remain unclear, but numerous studies have shown high ferritin expression in many cancer types. A large case–control study by Wang et al. found that there is a significant correlation between prostate cancer and serum ferritin, with ferritin serving as a non-invasive biomarker that could complement prostate-specific antigen testing in diagnosis and prognostic assessment [42]. Additionally, serum ferritin can serve as an independent prognostic biomarker for survival in patients with recurrent or refractory metastatic colorectal cancer [43].

Transferrin, as same as ferritin, is a biochemical marker for diagnosing blood iron homeostasis traditionally. It is an acute phase reactant and the primary plasma protein for iron transport. In organisms, transferrin exists in two forms: iron-unsaturated and iron-saturated. Ivanova et al. demonstrated the stimulatory effect of transferrin in the progression of advanced serous ovarian cancer [8]. Nevertheless, the role of transferrin has not been fully studied in the pathogenesis of malignant tumors. Transferrin saturation, indicating the availability of iron to tissues, serves as a marker of circulating iron [11]. Experimental studies indicate that increased iron storage in the body correlates with a higher overall cancer risk, particularly for liver, lung, colorectal, esophageal, gastrointestinal, and pancreatic cancers. Transferrin saturation levels exceeding 40% above normal iron reserves are linked to an elevated risk of cancer and mortality [12].

Iron is a crucial trace element for thyroid hormone synthesis, serving as a cofactor for thyroid peroxidase (TPO) and playing a significant role in the process of iodine oxidation catalyzed by TPO and thyroid hormone synthesis [48–50]. Iron deficiency can reduce the activity of TPO, thereby affecting the synthesis of thyroid hormones [49, 50]. Thyroid hormones can also influence iron balance by affecting the mRNA and protein expression of iron metabolism-related proteins, such as ferritin and transferrin [51]. The thyroid hormone L-thyroxine (T4) has been shown to stimulate the proliferation of various forms of cancer cells [60]. The connection between imbalances in iron metabolism and cancer development and tumorigenesis is gradually being recognized. Iron ions (especially ferrous iron Fe(2 +)) in iron metabolism can initiate the Fenton reaction, generating hydroxyl radicals that lead to oxidative stress and lipid peroxidation, which in turn affects DNA damage and cellular function [61]. Iron-induced lipid peroxidation has been linked to thyroid cancer [62]. Iron ions may also regulate the immune response and cell death in thyroid cancer by influencing the expression and function of specific proteins. For instance, NGAL (an iron-binding protein) is highly expressed in thyroid cancer and controls cell survival by inhibiting the FAS/CD95 death receptor [63]. When exploring the relationship between iron and thyroid cancer, it is essential to consider iron metabolism pathways in the liver and their impact on systemic iron levels, particularly the crucial role of hepcidin expression in regulating iron absorption and release. The liver’s regulation of iron may indirectly affect thyroid function by influencing iron distribution in other tissues [64].

Iron is an essential inorganic element for cell proliferation and growth, directly related to cell metabolism [52]. Due to their rapid growth rate, tumor cells require higher iron concentrations [53] and exhibit a strong dependence on iron to support their proliferation [54]. Iron participates in redox reactions, but its reactivity can also lead to the generation of reactive oxygen species (ROS). Therefore, tumor cells rely on further antioxidant mechanisms to resist this stress [55]. Iron overload may promote tumor initiation, proliferation, metastasis, and angiogenesis [56]. The results from our study underscore the importance of precise management of iron status, which should be clinically noted. Regular monitoring of iron levels in patients, especially for those at high risk of thyroid cancer (such as patients with a family history or previous radiation therapy), can help timely identify iron overload situations and take corresponding measures to reduce cancer risk. For instance, iron chelation therapy can be considered as a method to reduce iron load and strengthen the prevention of thyroid cancer [52]. Meanwhile, the relationship between iron and thyroid cancer may be realized through multiple biological mechanisms, including oxidative stress [57], activation of the NF-κB signaling pathway [58], regulation of cell proliferation [58], and influence on calcium ion homeostasis (59). Excessive iron not only increases free radical generation but also triggers chronic inflammatory responses, which may play a significant role in promoting the formation of the tumor microenvironment. Therefore, it is recommended to use antioxidants and anti-inflammatory drugs in clinical practice to help reduce the level of oxidative stress in the body, thereby mitigating its potential harm to thyroid cells. Since iron status is also closely related to the cell cycle and proliferation, individualized treatment strategies in clinical settings should consider the patient’s iron metabolism status. For example, patients with low iron status may affect thyroid hormone synthesis and thus their overall health, requiring comprehensive consideration during treatment to maintain a balance between iron and thyroid hormones. The results of this study indicate a close connection between iron status management and clinical intervention. Future research should aim to explore the complex interactions between iron and thyroid cancer, providing more evidence to support the evaluation and intervention of iron status.

Certain patients with TC are susceptible to early cervical lymph node metastasis, systemic metastasis, and tumor recurrence post-surgery and radiochemotherapy, resulting in poor prognoses [1]. Currently, effective early diagnostic markers and treatment methods for intervening in disease progression in these patients are lacking. Research highlights the key role of iron metabolism-related biomarkers in the development and progression of tumor cells. Serum ferritin has been identified as a reliable, convenient, and simple prognostic factor for survival [44]. It serves as a biomarker for head and neck carcinoma, lung carcinoma, renal cell carcinoma and pancreatic carcinoma, and as a susceptible marker for advanced tumor staging [45]. Additionally, study propose that transferrin could be a useful biomarker for the treatment and diagnosis of ovarian cancer [46]. Although our study did not find a correlation between transferrin and TC. Direct research on iron status as a clinically economical monitoring indicator for metastasis and recurrence in TC remains insufficient.

Our research demonstrates that the direction of the instrumental variable effect under models such as simple mode, weighted mode, and weighted median is consistent with the IVW model, enhancing the consistency and reliability of our conclusions. The alignment of multiple statistical models confirms a robust causal relationship between TC and iron status, bolstering the credibility of our findings. The Cochran’s Q test indicated that the three iron states did not exhibit significant heterogeneity in TC (Cochran’s Q *p-value* > *0.05*), suggesting no significant heterogeneity among different SNP loci. This supports the consistent effect of the selected instrumental variables, further enhancing the reliability of our conclusions. MR-Egger regression showed that the *P*-values of the intercepts for all indicators were greater than 0.05, with intercepts close to 0, indicating no influence of horizontal pleiotropy. This means the instrumental variables are unaffected by horizontal pleiotropy, allowing us to confidently infer a direct causal effect of iron status on TC. Through leave-one-out analysis, the overall effect remained stable after sequentially excluding each instrumental variable site; additionally, reverse MR analysis displayed that TC had no vital impact on iron status (*P* > *0.05*). These findings rule out reverse causality, suggesting that TC does not significantly alter an individual’s iron status, thus supporting our conclusion that iron status is a significant factor in increasing the risk of TC. Consequently, iron status-related biomarkers are wished to become new orientation for the treatment of TC, early diagnostic markers, and therapeutic interventions.

This study has some limitations, such as the absence of experimental validation, a relatively limited sample size, and a lack of clinical validation data. Additionally, batch differences between different datasets necessitate further optimization of the data processing workflow.

In summary, this study reveals a significant positive correlation between three iron states—Iron, Ferritin, and Transferrin Saturation—and TC through MR analysis. These findings supply a new viewpoint for understanding the disease and lay the foundation for future development of novel prevention and treatment strategies targeting iron metabolism pathways. Future research aims to incorporate more clinical data and experimental validation to further consolidate and expand these preliminary findings.

## Conclusion

Our mendelian study revealed a significant positive correlation between three iron states—iron, ferritin, and transferrin saturation—and thyroid cance, with no association found between transferrin and thyroid cancer. This provides new therapeutic strategies for the early diagnosis and treatment of thyroid cancer.

## Supplementary Information

Additional file1 (PDF 5 KB)

Additional file2 (PDF 5 KB)

Additional file3 (PDF 5 KB)

Additional file4 (PDF 5 KB)

Additional file5 (PDF 5 KB)

Additional file6 (PDF 5 KB)

Additional file7 (PDF 5 KB)

Additional file8 (PDF 5 KB)

Additional file9 (PDF 5 KB)

Additional file10 (PDF 98 KB)

Additional file11 (PDF 108 KB)

Additional file12 (PDF 112 KB)

Additional file13 (PDF 97 KB)

Additional file14 (PDF 108 KB)

Additional file15 (PDF 145 KB)

Additional file16 (DOCX 15 KB)

Additional file17 (DOCX 18 KB)

Additional file18 (DOCX 15 KB)

Additional file19 (DOCX 15 KB)

Additional file20 (CSV 1 KB)

## Data Availability

All data on the thyroid cancer and iron status that support the findings of this study are included within this paper and its Supplementary Information files. Example from: https://10.0.4.14/s41588-021-00931-x.
