# Supplementary figures and images for: A causal inference study exploring the impact of iron status on the risk of thyroid cancer based on two-sample mendelian randomization
Source: Discov Oncol. 2025 Apr 7;16:485. doi: 10.1007/s12672-025-02270-3 (PMC11977069; doi:10.1007/s12672-025-02270-3)

## MR Method

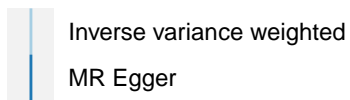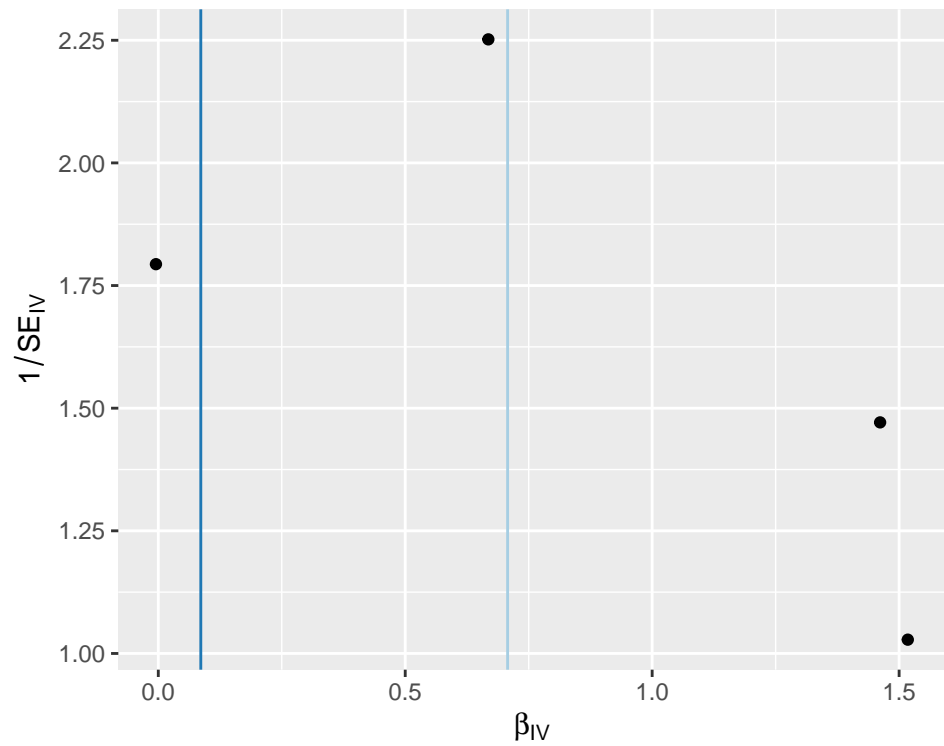

Supplement: Supplementary file 1 — Additional file1 (PDF 5 KB) [file 12672_2025_2270_MOESM1_ESM.pdf]

# MR Method

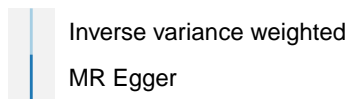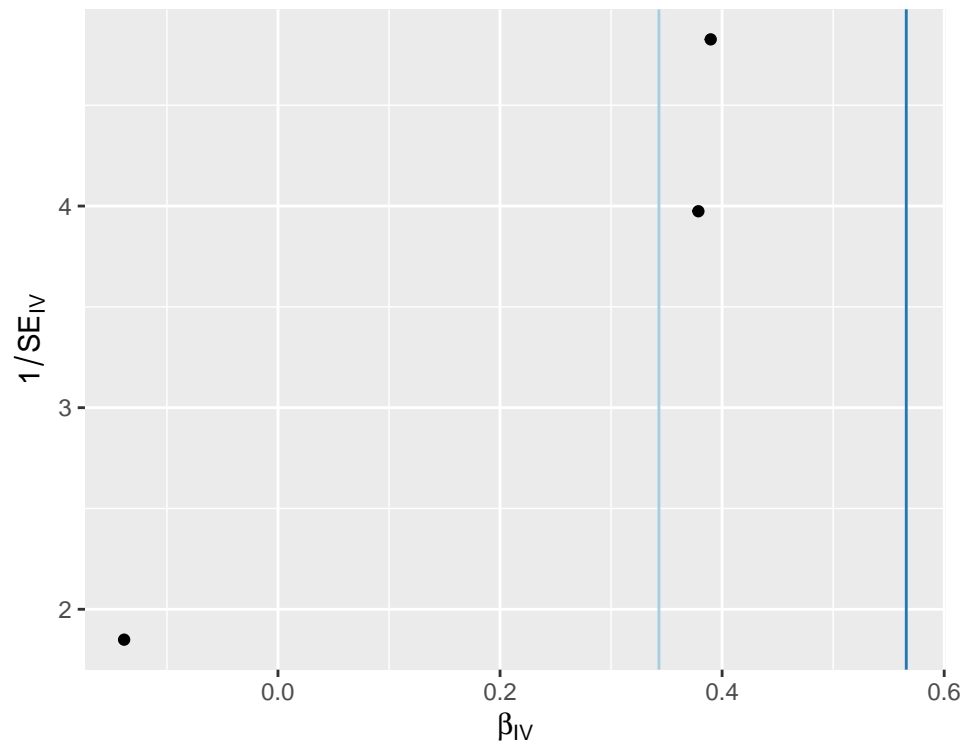

Supplement: Supplementary file 4 — Additional file4 (PDF 5 KB) [file 12672_2025_2270_MOESM4_ESM.pdf]

rs1525892

rs1800562

rs855791

All

0.00

0.25

0.50

0.75

MR leave-one-out sensitivity analysis for  
'Iron || id:ieu-a-1049' on 'Thyroid cancer || id:ebi-a-GCST90018929'

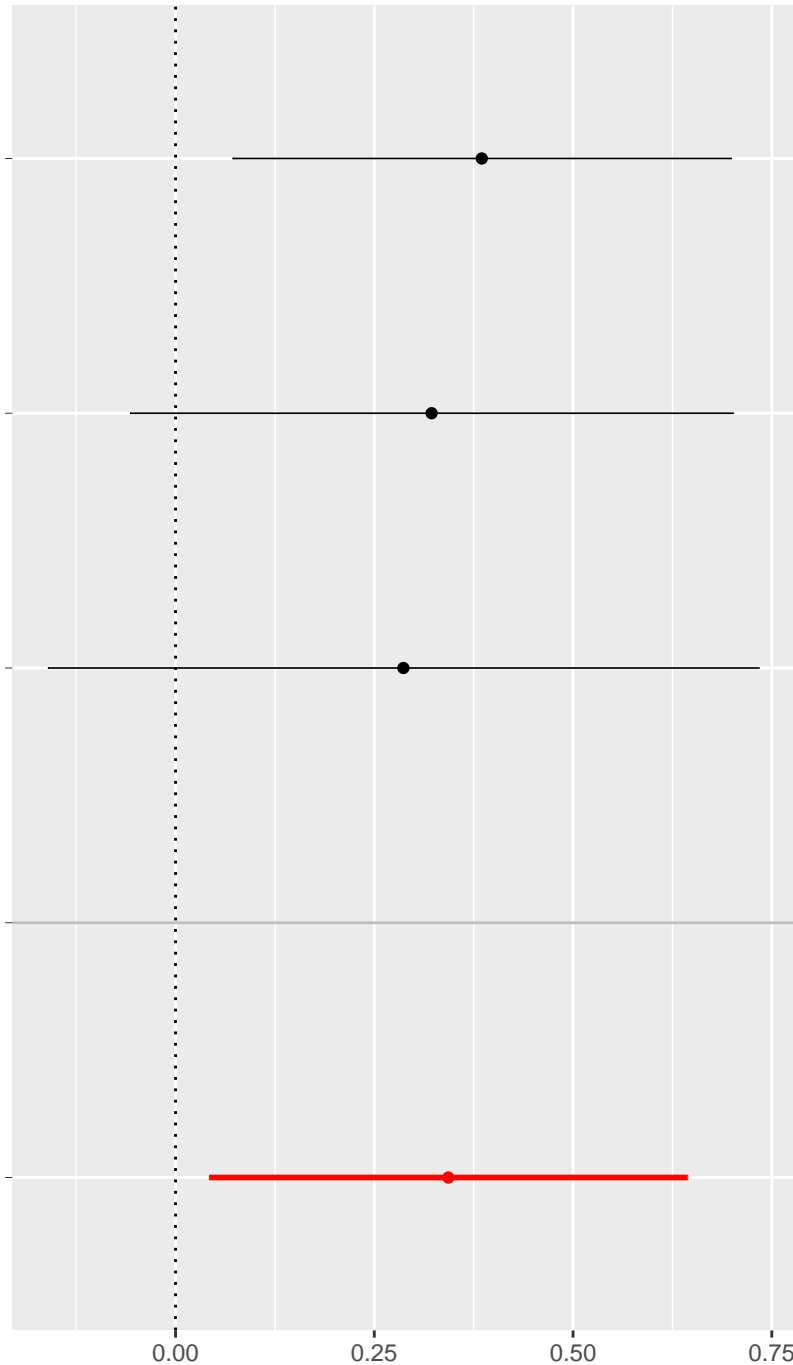

Supplement: Supplementary file 5 — Additional file5 (PDF 5 KB) [file 12672_2025_2270_MOESM5_ESM.pdf]

# MR Method

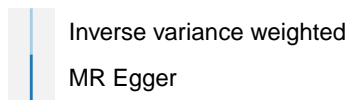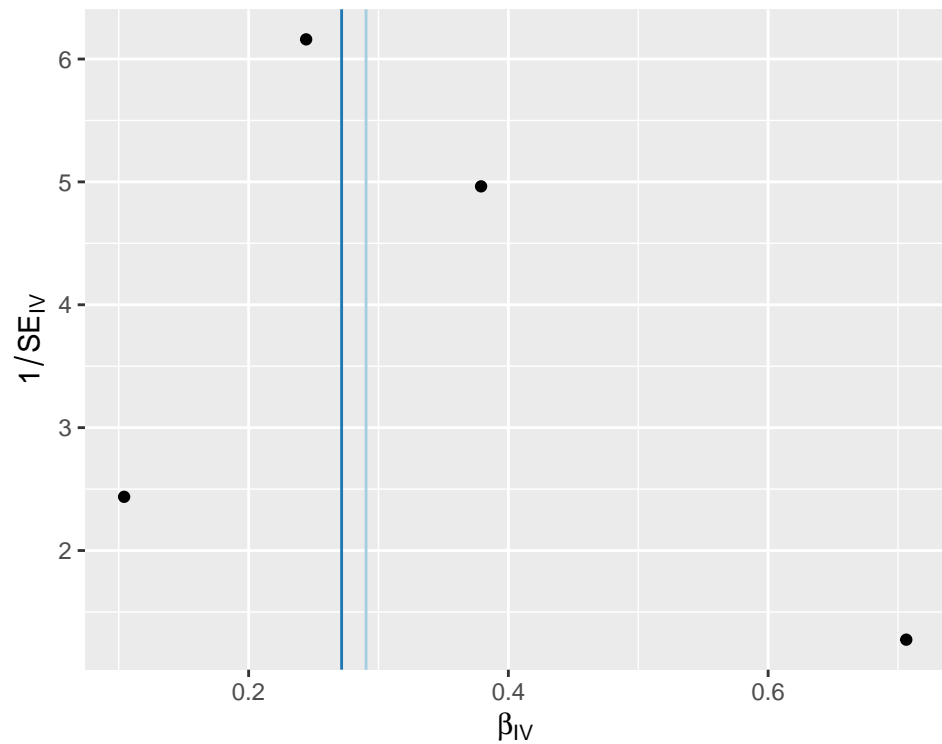

Supplement: Supplementary file 7 — Additional file7 (PDF 5 KB) [file 12672_2025_2270_MOESM7_ESM.pdf]

# MR Test

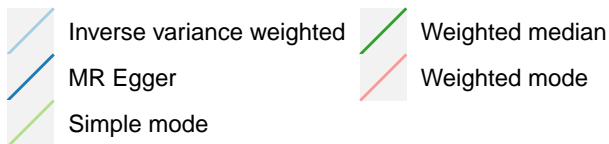

SNP effect on Thyroid cancer || id:ebi-a-GCST90018929

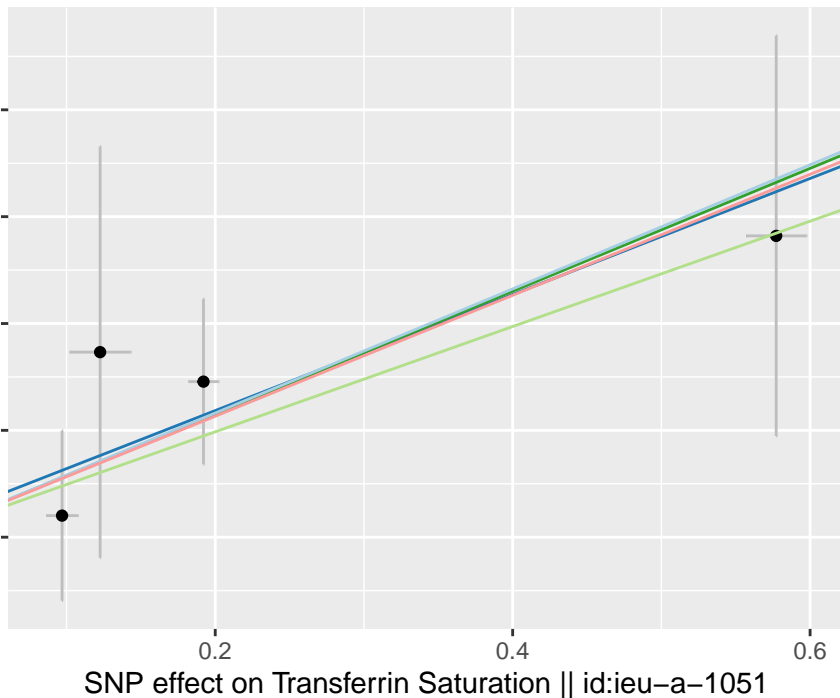

Supplement: Supplementary file 9 — Additional file9 (PDF 5 KB) [file 12672_2025_2270_MOESM9_ESM.pdf]

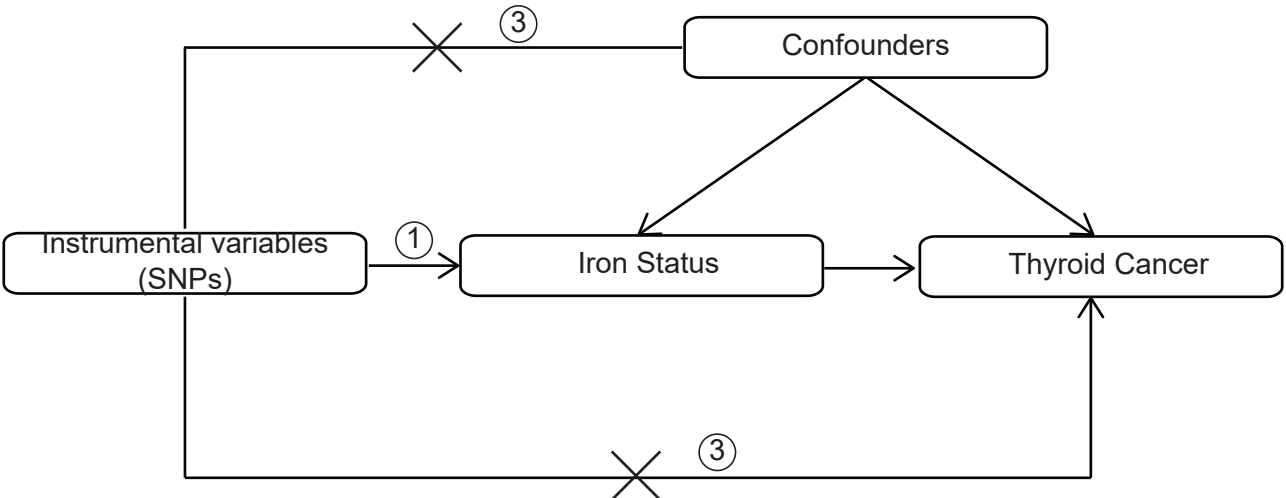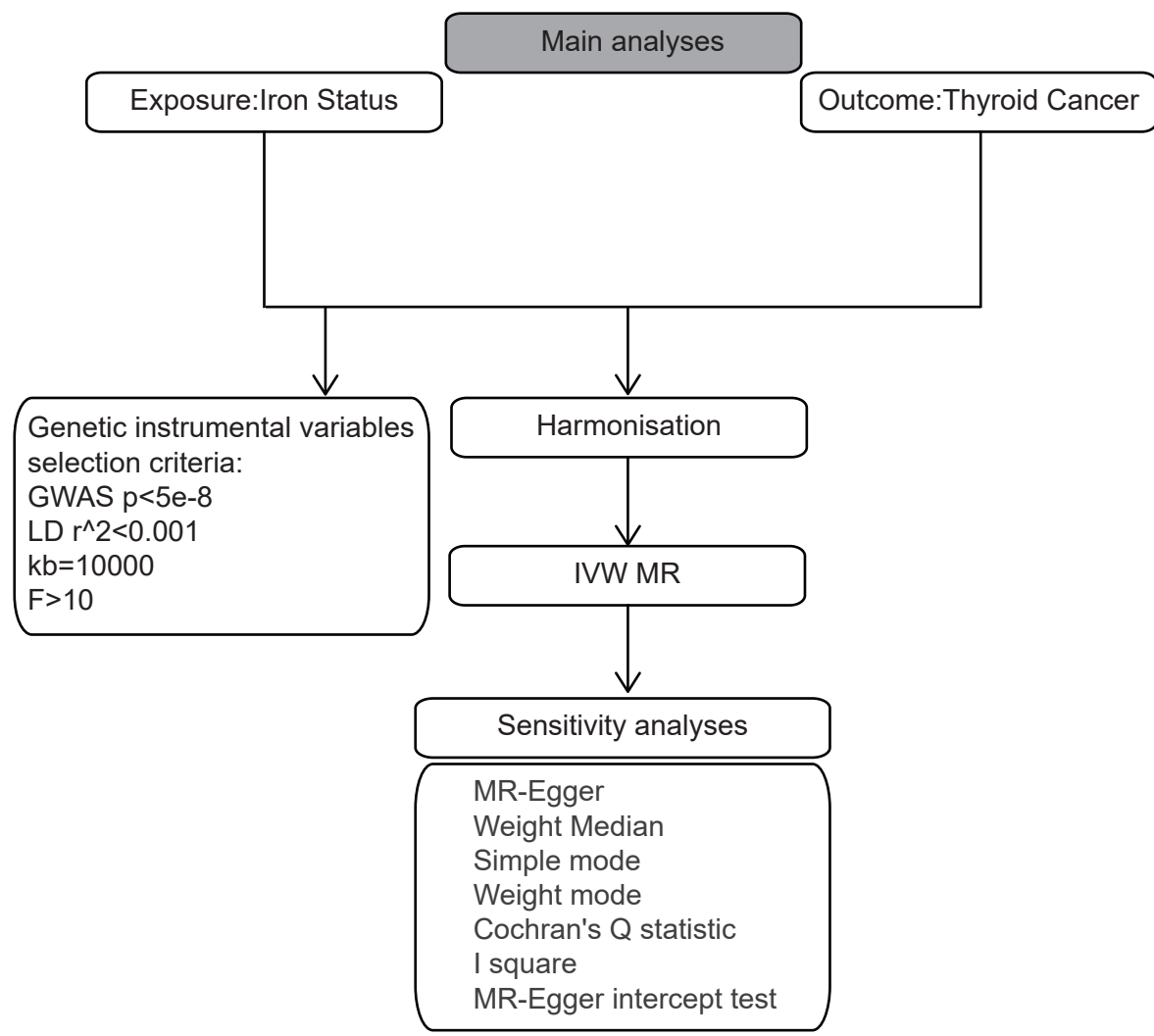

Supplement: Supplementary file 10 — Additional file10 (PDF 98 KB) [file 12672_2025_2270_MOESM10_ESM.pdf]

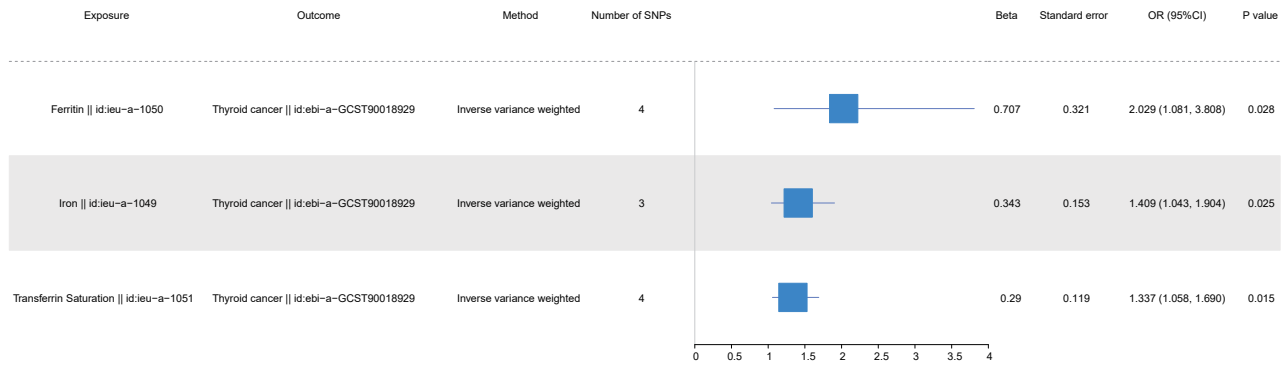

Supplement: Supplementary file 11 — Additional file11 (PDF 108 KB) [file 12672_2025_2270_MOESM11_ESM.pdf]

A

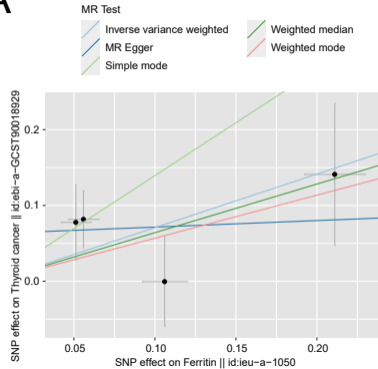

B

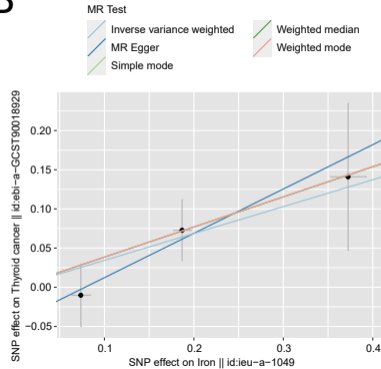

C

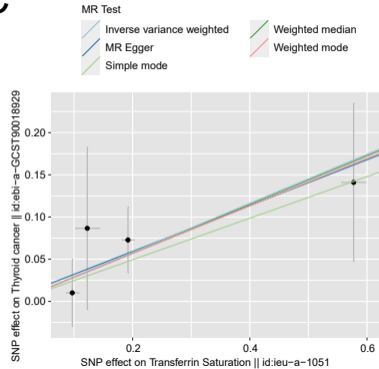

Supplement: Supplementary file 12 — Additional file12 (PDF 112 KB) [file 12672_2025_2270_MOESM12_ESM.pdf]

A

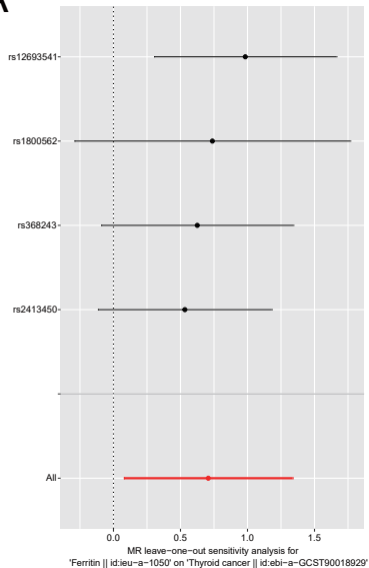

B

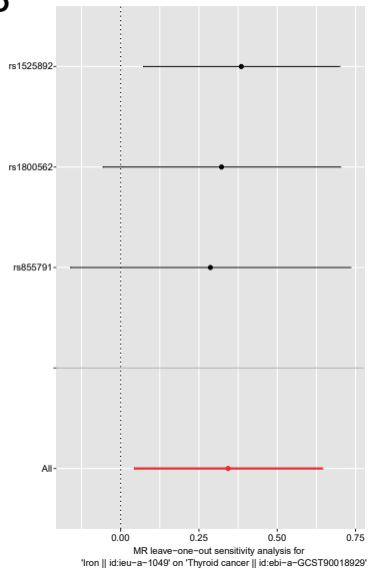

C

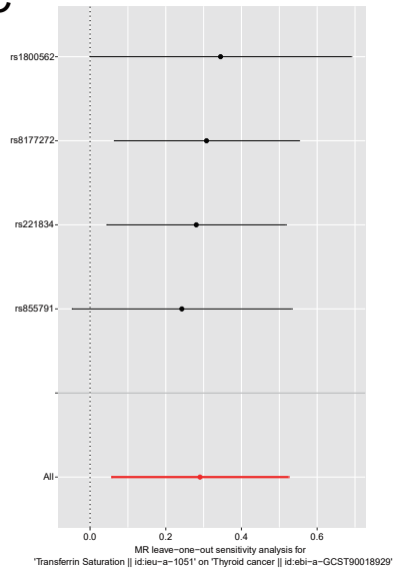

Supplement: Supplementary file 14 — Additional file14 (PDF 108 KB) [file 12672_2025_2270_MOESM14_ESM.pdf]
