# Supplementary material for: A causal inference study exploring the impact of iron status on the risk of thyroid cancer based on two-sample mendelian randomization
Source: Discov Oncol. 2025 Apr 7;16:485. doi: 10.1007/s12672-025-02270-3 (PMC11977069; doi:10.1007/s12672-025-02270-3)
Supplement: Supplementary file 2 — Additional file2 (PDF 5 KB) [file 12672_2025_2270_MOESM2_ESM.pdf]

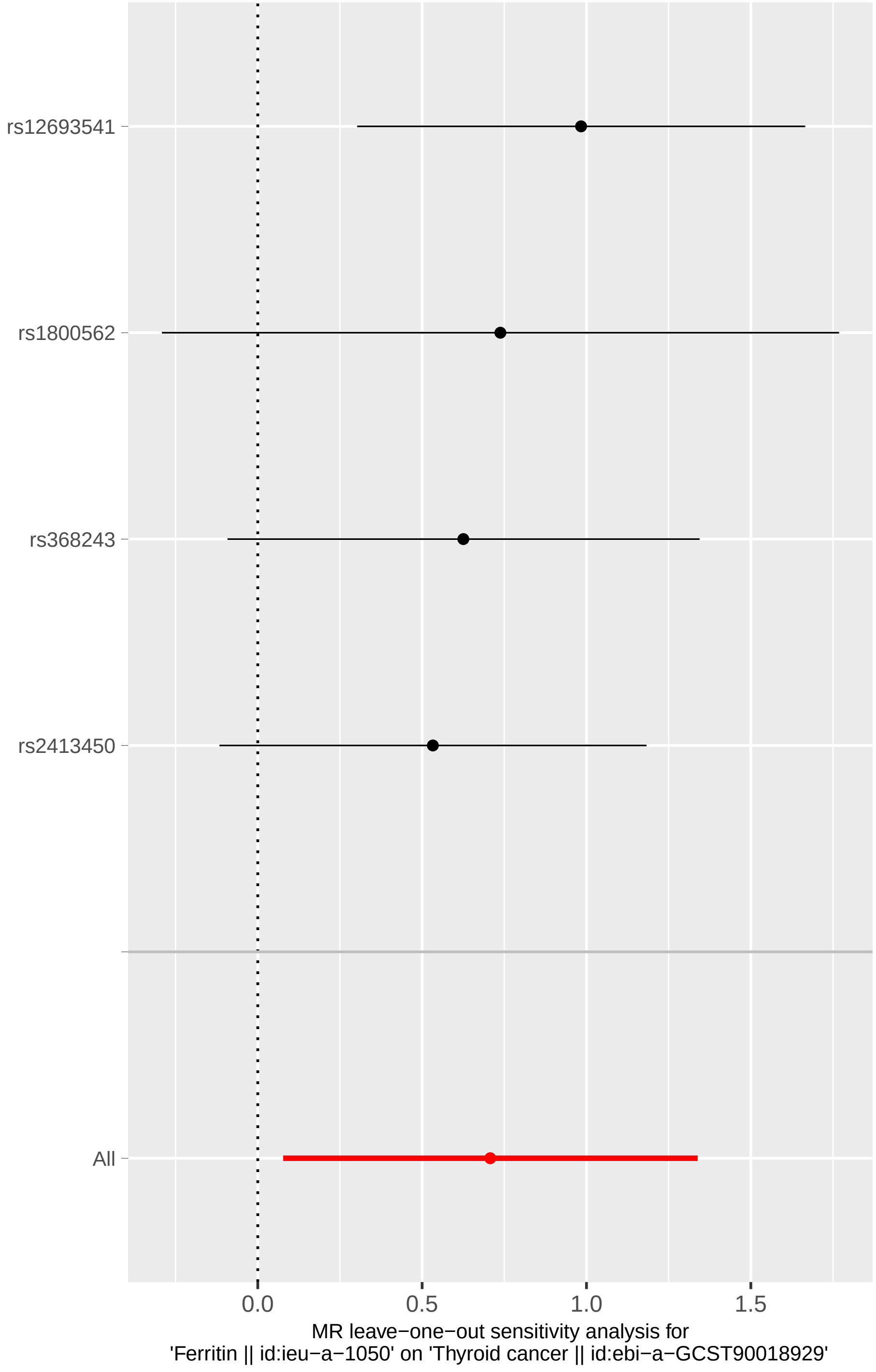
rs12693541
rs1800562
rs368243
rs2413450
All
0.0
0.5
1.0
1.5
MR leave−one−out sensitivity analysis for
'Ferritin || id:ieu−a−1050' on 'Thyroid cancer || id:ebi−a−GCST90018929'
