# Supplementary material for: A causal inference study exploring the impact of iron status on the risk of thyroid cancer based on two-sample mendelian randomization
Source: Discov Oncol. 2025 Apr 7;16:485. doi: 10.1007/s12672-025-02270-3 (PMC11977069; doi:10.1007/s12672-025-02270-3)
Supplement: Supplementary file 3 — Additional file3 (PDF 5 KB) [file 12672_2025_2270_MOESM3_ESM.pdf]

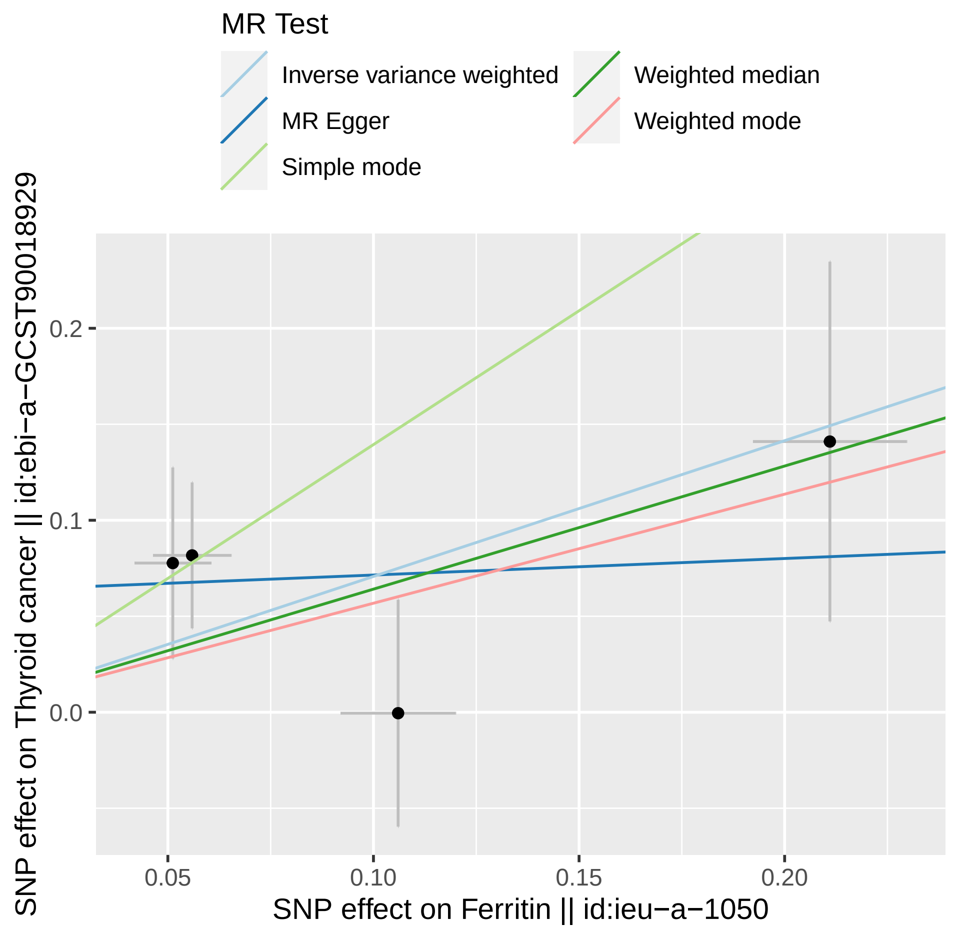

MR Test
Inverse variance weighted
MR Egger
Simple mode
Weighted median
Weighted mode
0.2
0.1
0.0
0.05
0.10
0.15
0.20
SNP effect on Ferritin || id:ieu−a−1050
SNP effect on Thyroid cancer || id:ebi−a−GCST90018929
