# Supplementary material for: A causal inference study exploring the impact of iron status on the risk of thyroid cancer based on two-sample mendelian randomization
Source: Discov Oncol. 2025 Apr 7;16:485. doi: 10.1007/s12672-025-02270-3 (PMC11977069; doi:10.1007/s12672-025-02270-3)
Supplement: Supplementary file 8 — Additional file8 (PDF 5 KB) [file 12672_2025_2270_MOESM8_ESM.pdf]

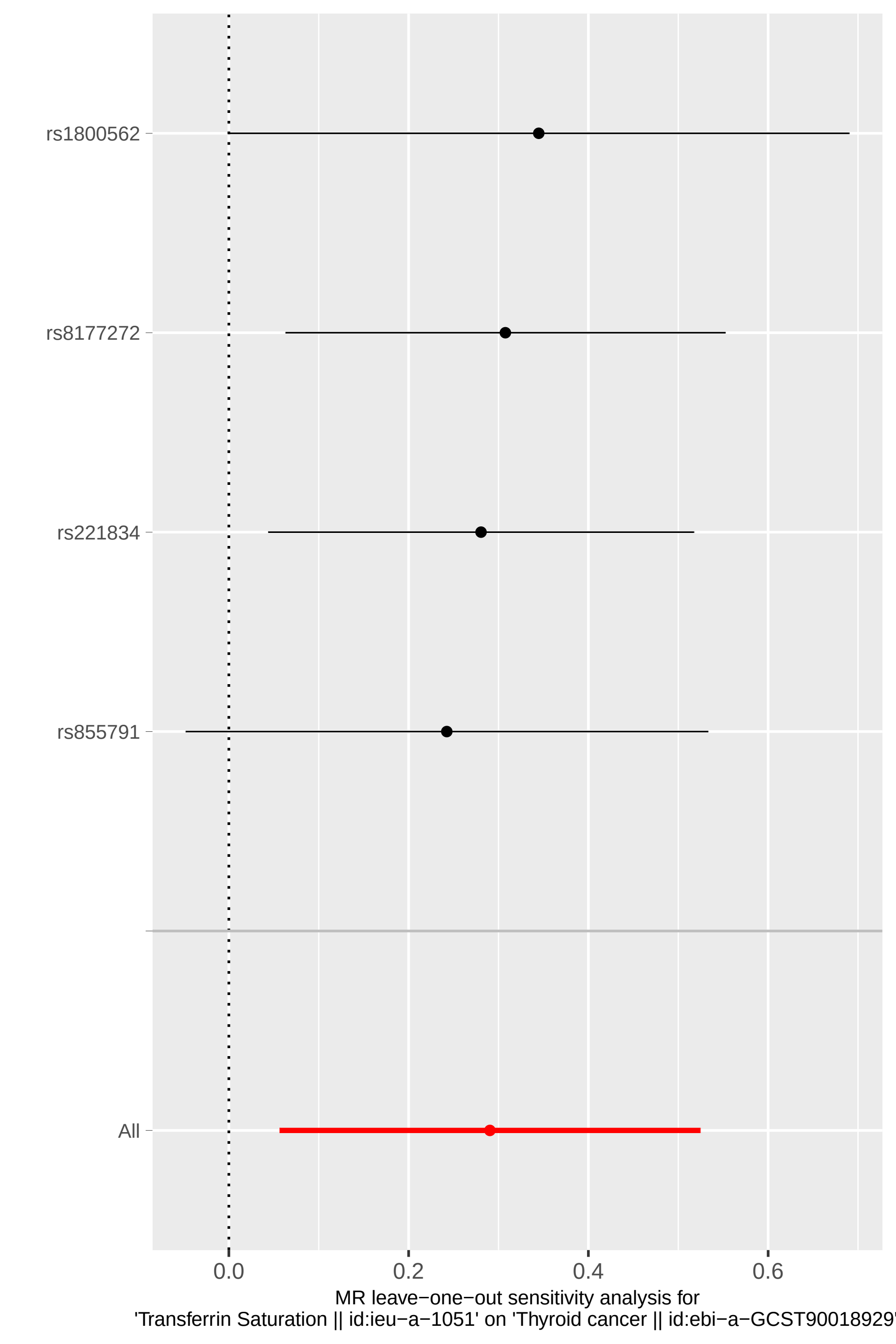
rs1800562
rs8177272
rs221834
rs855791
All
0.0
0.2
0.4
0.6
MR leave−one−out sensitivity analysis for
'Transferrin Saturation || id:ieu−a−1051' on 'Thyroid cancer || id:ebi−a−GCST90018929'
