# Supplementary material for: A causal inference study exploring the impact of iron status on the risk of thyroid cancer based on two-sample mendelian randomization
Source: Discov Oncol. 2025 Apr 7;16:485. doi: 10.1007/s12672-025-02270-3 (PMC11977069; doi:10.1007/s12672-025-02270-3)
Supplement: Supplementary file 13 — Additional file13 (PDF 97 KB) [file 12672_2025_2270_MOESM13_ESM.pdf]

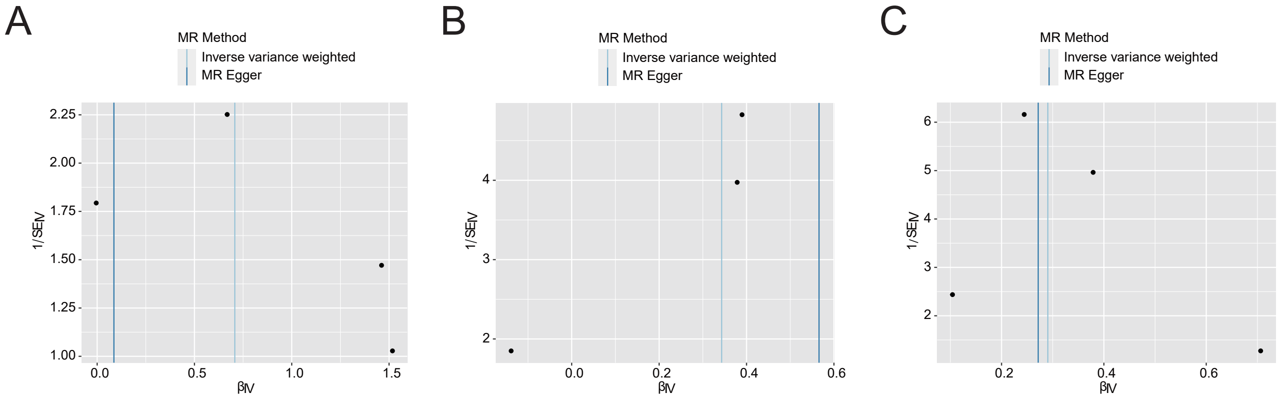

A
B
C
MR Method
Inverse variance weighted
MR Egger
1/SEIV
βIV
2.25
2.00
1.75
1.50
1.25
1.00
0.0
0.5
1.0
1.5
4
3
2
0.0
0.2
0.4
0.6
6
5
4
3
2
0.2
0.4
0.6
