# Supplementary material for: A causal inference study exploring the impact of iron status on the risk of thyroid cancer based on two-sample mendelian randomization
Source: Discov Oncol. 2025 Apr 7;16:485. doi: 10.1007/s12672-025-02270-3 (PMC11977069; doi:10.1007/s12672-025-02270-3)

Exposure	Outcome	Method	Number of SNPs	Beta	Standard error	OR (95%CI)	P value
Thyroid cancer || id:ebi−a−GCST90018929	Ferritin || id:ieu−a−1050	Inverse variance weighted	3	−0.007	0.015	0.993 (0.964, 1.023)	0.619
Thyroid cancer || id:ebi−a−GCST90018929	Ferritin || id:ieu−a−1050	MR Egger	3	0.011	0.040	1.011 (0.935, 1.094)	0.826
Thyroid cancer || id:ebi−a−GCST90018929	Ferritin || id:ieu−a−1050	Simple mode	3	−0.006	0.021	0.994 (0.954, 1.036)	0.816
Thyroid cancer || id:ebi−a−GCST90018929	Ferritin || id:ieu−a−1050	Weighted median	3	−0.005	0.016	0.995 (0.964, 1.027)	0.753
Thyroid cancer || id:ebi−a−GCST90018929	Ferritin || id:ieu−a−1050	Weighted mode	3	−0.004	0.017	0.996 (0.963, 1.030)	0.840
Thyroid cancer || id:ebi−a−GCST90018929	Iron || id:ieu−a−1049	Inverse variance weighted	3	−0.016	0.020	0.984 (0.946, 1.023)	0.439
Thyroid cancer || id:ebi−a−GCST90018929	Iron || id:ieu−a−1049	MR Egger	3	0.058	0.043	1.060 (0.974, 1.153)	0.406
Thyroid cancer || id:ebi−a−GCST90018929	Iron || id:ieu−a−1049	Simple mode	3	−0.052	0.036	0.949 (0.885, 1.019)	0.285
Thyroid cancer || id:ebi−a−GCST90018929	Iron || id:ieu−a−1049	Weighted median	3	−0.013	0.017	0.987 (0.955, 1.021)	0.422
Thyroid cancer || id:ebi−a−GCST90018929	Iron || id:ieu−a−1049	Weighted mode	3	−0.001	0.018	0.999 (0.964, 1.035)	0.981
Thyroid cancer || id:ebi−a−GCST90018929	Transferrin Saturation || id:ieu−a−1051	Inverse variance weighted	3	−0.013	0.020	0.987 (0.949, 1.027)	0.517
Thyroid cancer || id:ebi−a−GCST90018929	Transferrin Saturation || id:ieu−a−1051	MR Egger	3	0.058	0.043	1.060 (0.974, 1.153)	0.405
Thyroid cancer || id:ebi−a−GCST90018929	Transferrin Saturation || id:ieu−a−1051	Simple mode	3	−0.056	0.038	0.946 (0.878, 1.019)	0.278
Thyroid cancer || id:ebi−a−GCST90018929	Transferrin Saturation || id:ieu−a−1051	Weighted median	3	−0.015	0.016	0.985 (0.955, 1.016)	0.353
Thyroid cancer || id:ebi−a−GCST90018929	Transferrin Saturation || id:ieu−a−1051	Weighted mode	3	0.006	0.018	1.006 (0.971, 1.042)	0.758

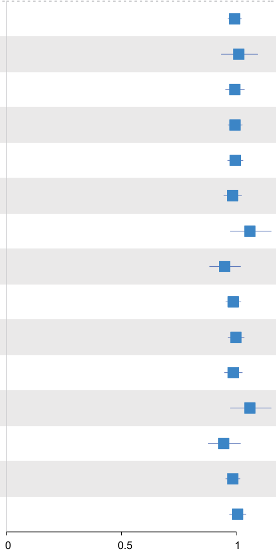

Supplement: Supplementary file 15 — Additional file15 (PDF 145 KB) [file 12672_2025_2270_MOESM15_ESM.pdf]
