# Supplementary material for: A causal inference study exploring the impact of iron status on the risk of thyroid cancer based on two-sample mendelian randomization
Source: Discov Oncol. 2025 Apr 7;16:485. doi: 10.1007/s12672-025-02270-3 (PMC11977069; doi:10.1007/s12672-025-02270-3)
Supplement: Supplementary file 16 — Additional file16 (DOCX 15 KB) [file 12672_2025_2270_MOESM16_ESM.docx]

**Table 1 铁状态和甲状腺癌的工具变量筛选及工具变量强度F检验**

**Table 1 Instrumental variable screening and instrumental variable strength F test for Iron Status and Thyroid Cancer.**

| exposure | outcome | Number of SNPs | Median of F | Minimum of F | Maximum of F |
| --- | --- | --- | --- | --- | --- |
| Ferritin \|\| id:ieu-a-1050 | Thyroid cancer \|\| id:ebi-a-GCST90018929 | 4 | 45.971 | 30.307 | 127.305 |
| Iron \|\| id:ieu-a-1049 | Thyroid cancer \|\| id:ebi-a-GCST90018929 | 3 | 342.038 | 50.079 | 346.675 |
| Transferrin Saturation \|\| id:ieu-a-1051 | Thyroid cancer \|\| id:ebi-a-GCST90018929 | 4 | 222.728 | 35.763 | 808.396 |

SNP，single nucleotide polymorphism.
