# Supplementary material for: A causal inference study exploring the impact of iron status on the risk of thyroid cancer based on two-sample mendelian randomization
Source: Discov Oncol. 2025 Apr 7;16:485. doi: 10.1007/s12672-025-02270-3 (PMC11977069; doi:10.1007/s12672-025-02270-3)
Supplement: Supplementary file 17 — Additional file17 (DOCX 18 KB) [file 12672_2025_2270_MOESM17_ESM.docx]

**Table 2 铁状态对甲状腺癌发病的孟德尔随机化分析结果**

**Table 2 Results of mendelian randomization analysis of Iron Status on the incidence of Thyroid Cancer**

| Exposure | Outcome | Method | Number of SNPs | β | Standard error | OR(95%CI) | P value |
| --- | --- | --- | --- | --- | --- | --- | --- |
| Ferritin \|\| id:ieu-a-1050 | Thyroid cancer \|\| id:ebi-a-GCST90018929 | Inverse variance weighted | 4 | 0.707 | 0.321 | 2.029 (1.081, 3.808) | 0.028 |
| Ferritin \|\| id:ieu-a-1050 | Thyroid cancer \|\| id:ebi-a-GCST90018929 | MR Egger | 4 | 0.086 | 0.605 | 1.090 (0.333, 3.567) | 0.9 |
| Ferritin \|\| id:ieu-a-1050 | Thyroid cancer \|\| id:ebi-a-GCST90018929 | Simple mode | 4 | 1.394 | 0.589 | 4.031 (1.271, 12.786) | 0.099 |
| Ferritin \|\| id:ieu-a-1050 | Thyroid cancer \|\| id:ebi-a-GCST90018929 | Weighted median | 4 | 0.641 | 0.387 | 1.899 (0.890, 4.052) | 0.097 |
| Ferritin \|\| id:ieu-a-1050 | Thyroid cancer \|\| id:ebi-a-GCST90018929 | Weighted mode | 4 | 0.568 | 0.441 | 1.765 (0.743, 4.189) | 0.288 |
| Iron \|\| id:ieu-a-1049 | Thyroid cancer \|\| id:ebi-a-GCST90018929 | Inverse variance weighted | 3 | 0.343 | 0.153 | 1.409 (1.043, 1.904) | 0.025 |
| Iron \|\| id:ieu-a-1049 | Thyroid cancer \|\| id:ebi-a-GCST90018929 | MR Egger | 3 | 0.566 | 0.313 | 1.761 (0.954, 3.251) | 0.321 |
| Iron \|\| id:ieu-a-1049 | Thyroid cancer \|\| id:ebi-a-GCST90018929 | Simple mode | 3 | 0.384 | 0.199 | 1.469 (0.995, 2.169) | 0.193 |
| Iron \|\| id:ieu-a-1049 | Thyroid cancer \|\| id:ebi-a-GCST90018929 | Weighted median | 3 | 0.384 | 0.156 | 1.469 (1.082, 1.994) | 0.014 |
| Iron \|\| id:ieu-a-1049 | Thyroid cancer \|\| id:ebi-a-GCST90018929 | Weighted mode | 3 | 0.385 | 0.184 | 1.470 (1.025, 2.110) | 0.172 |
| Transferrin Saturation \|\| id:ieu-a-1051 | Thyroid cancer \|\| id:ebi-a-GCST90018929 | Inverse variance weighted | 4 | 0.29 | 0.119 | 1.337 (1.058, 1.690) | 0.015 |
| Transferrin Saturation \|\| id:ieu-a-1051 | Thyroid cancer \|\| id:ebi-a-GCST90018929 | MR Egger | 4 | 0.272 | 0.21 | 1.312 (0.870, 1.979) | 0.325 |
| Transferrin Saturation \|\| id:ieu-a-1051 | Thyroid cancer \|\| id:ebi-a-GCST90018929 | Simple mode | 4 | 0.246 | 0.165 | 1.279 (0.927, 1.766) | 0.231 |
| Transferrin Saturation \|\| id:ieu-a-1051 | Thyroid cancer \|\| id:ebi-a-GCST90018929 | Weighted median | 4 | 0.288 | 0.126 | 1.333 (1.041, 1.708) | 0.023 |
| Transferrin Saturation \|\| id:ieu-a-1051 | Thyroid cancer \|\| id:ebi-a-GCST90018929 | Weighted mode | 4 | 0.283 | 0.144 | 1.327 (1.001, 1.759) | 0.144 |

β，Mendelian randomization analysis effect coefficient；OR, odds ratio；CI，confidence interval； SNP， single nucleotide polymorphism；MR，mendelian randomization.
