# Supplementary material for: A causal inference study exploring the impact of iron status on the risk of thyroid cancer based on two-sample mendelian randomization
Source: Discov Oncol. 2025 Apr 7;16:485. doi: 10.1007/s12672-025-02270-3 (PMC11977069; doi:10.1007/s12672-025-02270-3)
Supplement: Supplementary file 18 — Additional file18 (DOCX 15 KB) [file 12672_2025_2270_MOESM18_ESM.docx]

**Table 3 铁状态对甲状腺癌发病的孟德尔随机化分析的Cochran Q检验异质性**

**Table 3 Heterogeneity of the Cochran Q test for mendelian randomization analysis of Iron Status on the incidence of Thyroid Cancer.**

| Exposure | Outcome | Method | Cochran Q | Cochran Q df | Cochran Q p-value | I2 (%) |
| --- | --- | --- | --- | --- | --- | --- |
| Iron \|\| id:ieu-a-1049 | Thyroid cancer \|\| id:ebi-a-GCST90018929 | Inverse variance weighted | 0.864026 | 2 | 0.649201 | 0 |
| Ferritin \|\| id:ieu-a-1050 | Thyroid cancer \|\| id:ebi-a-GCST90018929 | Inverse variance weighted | 3.563626 | 3 | 0.312603 | 15.81607 |
| Transferrin Saturation \|\| id:ieu-a-1051 | Thyroid cancer \|\| id:ebi-a-GCST90018929 | Inverse variance weighted | 0.761118 | 3 | 0.858743 | 0 |

Q, Cochran Q test statistic; Q df, Q tests the degrees of freedom;MR，mendelian randomization.
