# Supplementary material for: A causal inference study exploring the impact of iron status on the risk of thyroid cancer based on two-sample mendelian randomization
Source: Discov Oncol. 2025 Apr 7;16:485. doi: 10.1007/s12672-025-02270-3 (PMC11977069; doi:10.1007/s12672-025-02270-3)
Supplement: Supplementary file 19 — Additional file19 (DOCX 15 KB) [file 12672_2025_2270_MOESM19_ESM.docx]

**Table 4 孟德尔随机化分析铁状态对甲状腺癌发病率影响的水平多效性检验**

**Table 4 Horizontal pleiotropy test for mendelian randomization analysis of Iron Status on the incidence of Thyroid Cancer.**

| Exposure | Outcome | MR-Egger intercept | Standard error | P value |
| --- | --- | --- | --- | --- |
| Iron \|\| id:ieu-a-1049 | Thyroid cancer \|\| id:ebi-a-GCST90018929 | -0.04434 | 0.054259 | 0.563853 |
| Ferritin \|\| id:ieu-a-1050 | Thyroid cancer \|\| id:ebi-a-GCST90018929 | 0.062858 | 0.05306 | 0.357846 |
| Transferrin Saturation \|\| id:ieu-a-1051 | Thyroid cancer \|\| id:ebi-a-GCST90018929 | 0.004919 | 0.045022 | 0.922977 |

MR-Egger ，mendelian randomization-Egger.
